# A green compliant hand-held selective electrode device for monitoring active pharmaceuticals and the kinetics of their degradation

**DOI:** 10.1038/s41598-023-38416-y

**Published:** 2023-07-21

**Authors:** Norhan Badr ElDin, Eslam Dabbish, Esraa Fawaz, Mohamed K. Abd El-Rahman, Tamer Shoeib

**Affiliations:** 1 Analytical Chemistry Department, Faculty of Pharmacy, Cairo University, Kasr-El Aini Street, Cairo, 11562 Egypt; 2 Department of Chemistry, The American University in Cairo, New Cairo, 11835 Egypt; 3 Department of Chemistry and Chemical Biology, Harvard University, 12 Oxford Street, Cambridge, MA 02138 USA

**Keywords:** Analytical chemistry, Electrochemistry

## Abstract

An *in-line* smartphone connected to a screen-printed selective electrode hand-held device was used to determine the concentration of distigmine bromide (DB) in its pure and dosage forms as well as its degradation kinetics by continuously measuring the change in the produced emf over time. The main objective, supported by the data presented, is to produce a highly reliable smartphone integrated selective sensor as a portable analyzer with potential high cloud connectivity combining a wide linear dynamic range, the fastest response time with the lowest limits of detection and quantitation while best integrating green analytical chemistry principles. The choice of ionophore used in this approach was guided by computation and the data obtained was compared with traditional analytical techniques. DB, for which there are no previously reported stability-indicating methods and for which four novel such methods are proposed here, was selected as a model drug for this work.* At-line* UV-spectrophotometry DB assay was obtained by measuring the difference between the spectra of the degradation product and the same concentration of intact drug. The degradation kinetics were studied by this method through tracking the decrease of DB absorbance and/or the increase of a generated degradation product signal over time. *Off-line* separation based HPLC and TLC stability-indicating methods for DB were also presented. All methods employed in this work were validated for accuracy, precision, specificity, repeatability, linearity, range, detection and quantification limits according to the ICH guidelines and were applied to the analysis of laboratory prepared mixtures as well as commercial products. While all methods proposed were shown to be highly reliable, the smartphone integrated selective sensor is highlighted as a portable analyzer with potential high cloud connectivity and was shown to combine a wide linear dynamic range, the fastest response time with the lowest limits of detection and quantitation while best integrating green analytical chemistry principles.

## Introduction

Significant efforts over the past few decades have been directed toward the adoption of green chemistry concepts into analytical measurements^1–5^. The large number of analytical instruments in use worldwide provide considerable motivational power to find alternatives that afford better environmental protection and sustainable use^6–8^. This may be partly attributed to the large quantities of hazardous substances that are used in different stages of the chemical analysis life-cycle, energy consumption and operator safety^9,10^. Consequently, green analytical chemistry concepts should be central in selecting and developing analytical methods^11^.

Recent advances in real-time monitoring and miniaturization of analyzers are recently explored to produce portable greener analytical instruments^12–18^. The implementation of such analyzers typically, significantly reduces lengthy sample preparation steps and large amounts of solvent and waste, thus decreasing the negative environmental consequences and improving decision-making. Analytical chemistry techniques that make use of these smarter and more sustainable technologies will therefore provide potential alternatives to conventional methods to meet future demands. Liquid, gas and thin layer chromatography as well as spectrophotometry are well-established, efficient and conventionally adopted in benchtop analytical techniques; however, they fall short in real-time data acquisition, economic operation, and being adaptable for miniaturization. This highlights the need for low-cost, highly accurate and sensitive, portable analytical tools affording real time measurements^19–22^. While some instruments may allow real time measurements such as micro-LC^23^, portable spectrometers^24^ and microfluidic devices^25^, however, there are no reports on the use of such devices in drug assay and the analysis of active pharmaceutical ingredients stability with the aim of monitoring their degradation kinetics. For these reasons, probe techniques remain in high demand thanks to the excellent characteristics shown by potentiometric sensors as portable and reliable analytical tools that can be fully integrated with other electronic devices to interpret their responses for in-line monitoring, for example, of the degradation kinetics of organic compounds^26–28^.

The unique abilities of ISEs for in-line measurements is the key driver for implementation of novel strategies to provide alternative, more environmentally friendly technologies, that could convey comparable analytical results to other traditional off-line techniques^29–33^. Currently, the market of portable devices, especially wearable ones, that can monitor many parameters, chiefly making use of electrochemical sensors, is rapidly expanding^34^.

The marriage of smartphone technology with potentiometric devices is thus a true departure from traditional analytical techniques, towards portable, cost-effective, real-time sensing approaches with high potential of rapid dissemination through internet and cloud sharing^35–39^.

**Figure 1:**
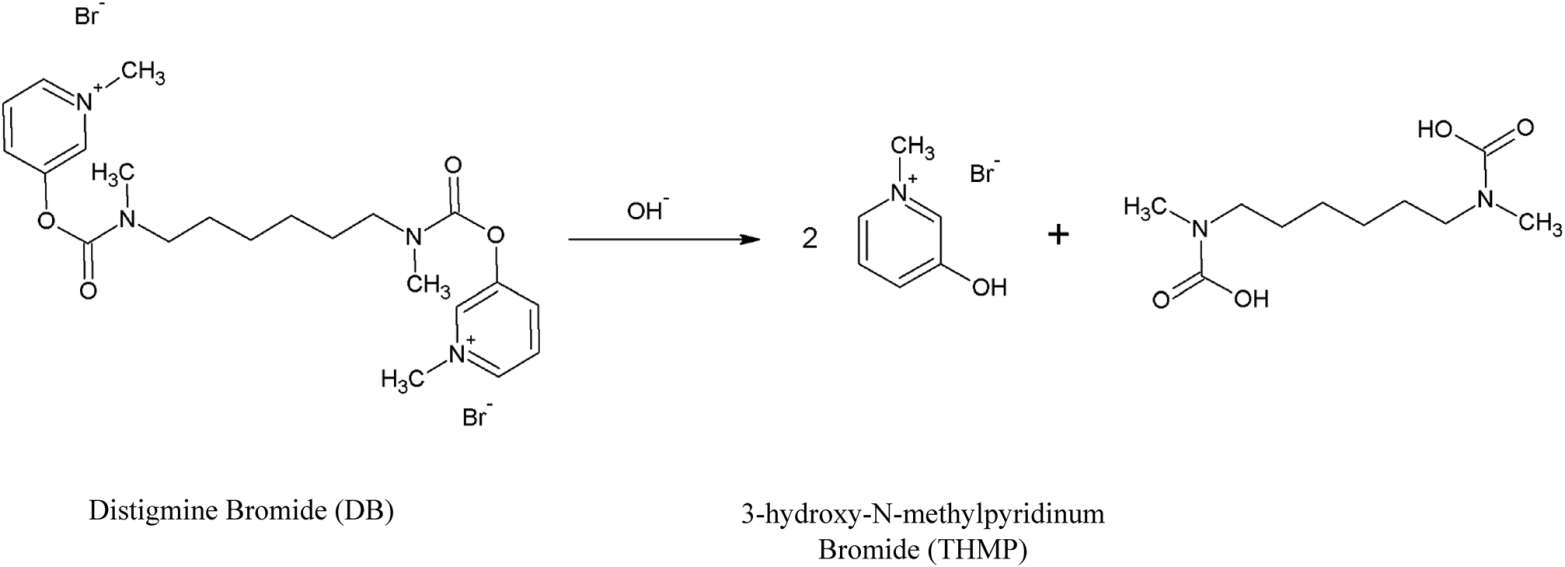
Alkaline hydrolysis of Distigmine Bromide (DB) to produce its degradation product THMP.

Believing in these growing opportunities, this study focuses upon developing a portable smartphone integrated potentiometric sensor for tracking the degradation kinetics of easily hydrolysable ionizable/ionic drugs. DB, shown in Fig. 1, was selected as a model drug based on its degradable carbamate linkage and di-quaternary ammonium groups, making it a strong cationic electrolyte. The choice of this drug is additionally justified by the unique pattern of transformation of its absorption spectrum to that of its degradation product, which may be tracked by continuous spectrophotometric scanning. In addition, the different lipophilicities of DB and its degradation product, which facilitate their chromatographic separations; and the fact that there are no reported stability indicating methods for its determination in spite of its longstanding commercial distribution and use, are all contributing factors in its selection.

In this work, four newly developed validated stability indicating methodologies, smartphone-based ISE, UV-Spectrophotometry, HPLC, and TLC-densitometry, for the determinations of DB in the presence of its alkaline degradation products, 3-hydroxy-*N*-methylpyridinum (THMP) were developed and presented. Data for hydrolysis rate constant and the hydrolysis activation energy were also reported. Finally, we provide a side-by-side comparison of the four analytical approaches to evaluate their accuracy and precision as well as their environmental sustainability by applying three assessment tools; being, the Green Analytical Procedure Index (GAPI)^40^, the Analytical Eco-Scale (ESA)^41^ and the Analytical Greenness Metric Approach (AGREE)^42^.

## Results and discussion

### Methods development

#### ISE membrane optimization

To improve the selectivity and LOD of the developed sensor^43,44^, the ion-selective membrane responsible for the sensing mechanism was impregnated with a lipophilic ion-exchanger and an ionophore. The former in this case was potassium tetrakis 4-chlorophenyl borate (KTCPB) that selectively includes distigmine di-cations (DG) in the organic layer and excludes the counter ions into the bulk aqueous phase, resulting in interfacial charge separation. Strong intermolecular association and complexation energies between the selected ionophore and DG as well as a presence of a fourfold molar excess of the ionophore relative to DG would reduce the concentration of free DG and thus, in turn lowers the rate of DG transfer across the membrane. This strong intermolecular association may also allow the DG within the complex with the chosen ionophore to exhibit a higher lipophilic character than it would as a free molecule. This would also lower transmembrane DG flux, thus improve signal stability, sensitivity and increase the life span of the membrane sensor produced. Two potential molecular receptors and ionophores for the DG were considered, these were non-hydroxylated calixarene 6, NCX6 and hydroxylated calixarene 6, CX6, the latter we have previously shown to be successfully used as an ionophore for the determination of singly positive choline ions in milk^18^. DFT calculations were employed to elucidate the structure of DG, the structures of both ionophores, as well as structures of their monomeric and dimeric complexes with DG. Calculations were also employed to detail the types of binding involved, provide values binding energies as well as to calculate the $logP$ values of the free species and the associated complexes.

#### Ionophore selection

The calculated most stable conformations of the two potential ionophores NCX6 and CX6 as well as their molecular electrostatic potential surfaces are shown in Figure S1 of the supplementary material. Both NCX6 and CX6 are characterized by cavities that are conical and non-symmetric. This cavity is stabilized in the case of CX6 by five hydrogen bonds between hydrogen atoms and phenolic oxygen atoms as detailed in Figure S1. The most stable conformation of DG and the corresponding molecular electrostatic potential surface clearly showing the two electron deficient sites on either end of the extended DG molecule are shown in Panels A and B in Figure S2 of the supplementary material, respectively.

**Figure 2:**
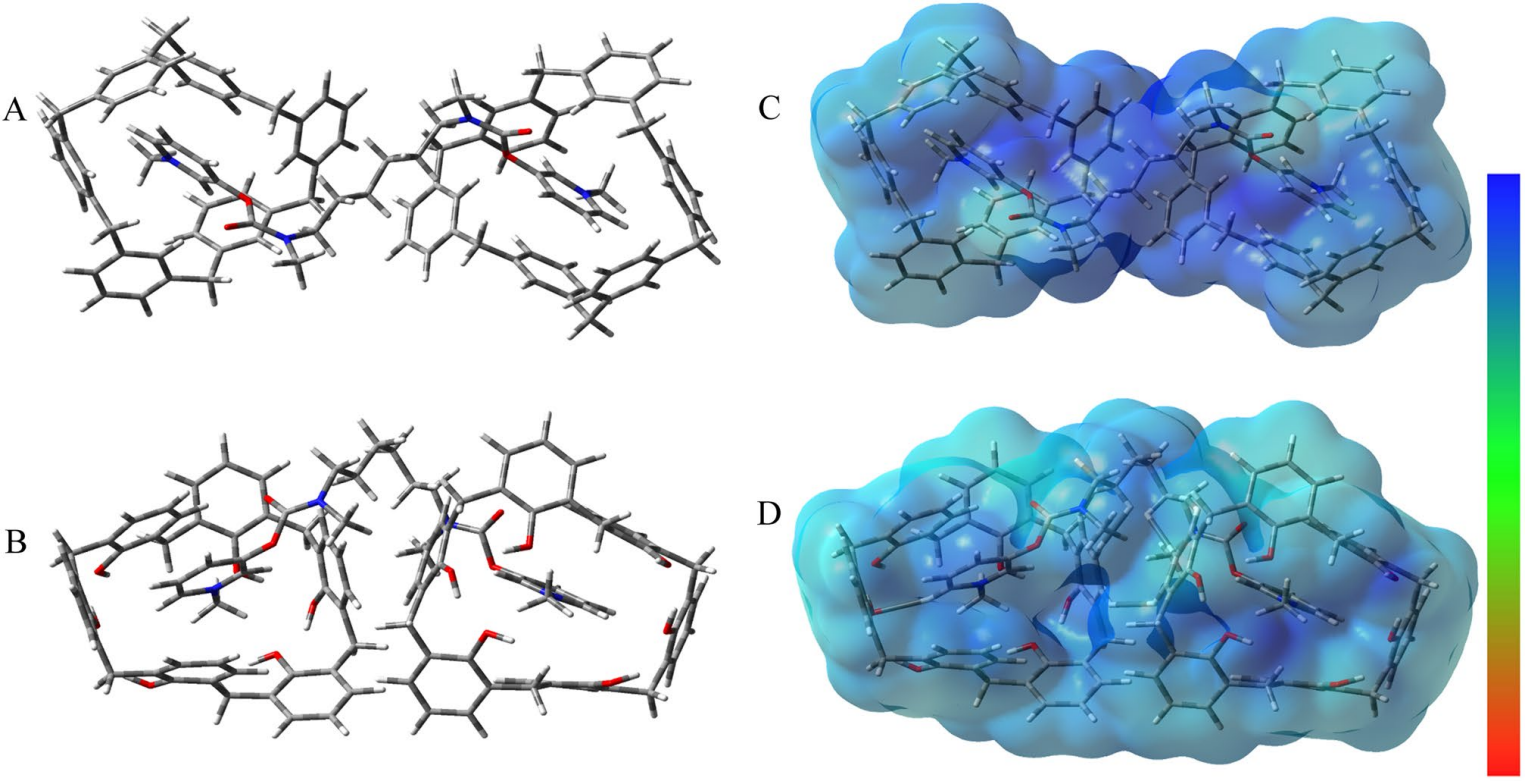
Panels (**A** and **B**) show optimized geometrical structures for 2:1 DG complexes with NCX6 and CX6 respectively as obtained using the B97D/6-31G(d,p) level of theory (6-31 + G(d,p) was used for oxygen atoms). Panels (**C** and **D**) show molecular electrostatic potential surfaces for the DG complexes with NCX6 and CX6 respectively as obtained from the gas phase optimized structures using cubegen utility in the Gaussian 16 package. The color code indicates electron rich (red) and electron deficient sites (blue).

Figure S3 of the supplementary material, shows the most stable 1:1 DG-NCX6 and DG-CX6 complexes and their corresponding molecular electrostatic potential surfaces. In either case one of the cationic pyridinium rings of DG is being hosted within the cavities of NCX6 or CX6. This lead to the delocalization of the positive charge of the complexed pyridinium rings as is evident in panels C and D of Figure S3 as well as the expansion and deformation of the cavities in these molecular hosts. In the case of CX6, this cavity expansion is also accompanied by the reduction in the number and the elongation of the remaining intramolecular hydrogen bonds. Both the 1:1 DG-NCX6 and DG-CX6 complexes are stabilized by cation-π interactions between the pyridinium tertiary nitrogen atoms acting as a π acceptors and the surrounding π cloud of the calixarene aromatic rings acting as the π donors. These interactions are evidenced through a comparison of the plotted non-covalent interactions and the reduced gradient plots in Figures S4 and S5. The calculated Gibbs binding free energies of complexation in a 1-nitro propane medium for the 1:1 DG-NCX6 and DG-CX6 species were determined to be − 15.8 and − 13.6 kcal mol^−1^ respectively. These values obtained in a 1-nitro propane medium which closely approximates the dielectric constant of 2-nitro phenyl octyl ether, the medium used experimentally to dissolve the ionophores, show that both of these complexes are stable and relatively close in binding free energy with a difference of 2.2 kcal mol^−1^. However, since a fourfold molar excess of the chosen ionophore relative to DG would be used in order to minimize the concentration of free DG and thus, in turn lowers the rate of DG transfer across the membrane calculations on 1:2 DG-NCX6 and DG-CX6 complexes were performed. These calculations show that in both cases, the lowest conformation of the 1:2 complexes formed, have the two cationic pyridinium rings of DG, each inserted in the cavity of a separate host to produce near symmetrical host-gest complexes. The geometries for the 1:2 DG-NCX6 and DG-CX6 complexes are highlighted in Panels A and B respectively in Fig. 2. The corresponding molecular electrostatic potential surfaces presented in Panels C and D in Fig. 2; on the other hand, show the distribution of the positive charge over the structure as a result of host–guest complexation. The non-covalent character of the interactions in these 1:2 DG complexes with either NCX6 and CX6 are clearly shown in Figure S6 of the supplementary materials. Figure S6 shows in both complexes, each of the pyridinium rings to be surrounded by six aromatic rings from different directions, thus these cation-π interaction can be identified in different binding fashions of edge to face and face to face. These interactions are significant as supported by the calculated Gibbs binding free energy for the 1:2 DG-NCX6 and DG-CX6 complexes obtained in a 1-nitro propane medium to be − 30.67 and − 30.24 kcal mol^−1^ respectively. In general, cation-π interactions ranging from − 12 to − 65 kcal mol^−1^ are reported^45^. This further indicates that these non-covalent guest–host interactions play a significant role in stabilizing the resulting complexes and play a part in reducing the concentration of free DG and thus, in turn inhibits the rate of DG transfer across the membrane. These 1:2 DG-NCX6 and DG-CX6 complexes also exhibited a significantly higher lipophilic character than that of free DG as demonstrated by the calculated $logP$ values of 9.98 and 7.24 for the two complexes respectively relative to − 3.55 for DG. This also is expected to lower transmembrane DB flux thus improving the signal stability, sensitivity and life span of the membrane sensor produced and clearly demonstrates the suitability of both ionophores. In this work we have adopted CX6 as the ionophore of choice due to its commercial availability and lower cost.

### *In-line* smartphone-based ISE measurements

**Figure 3:**
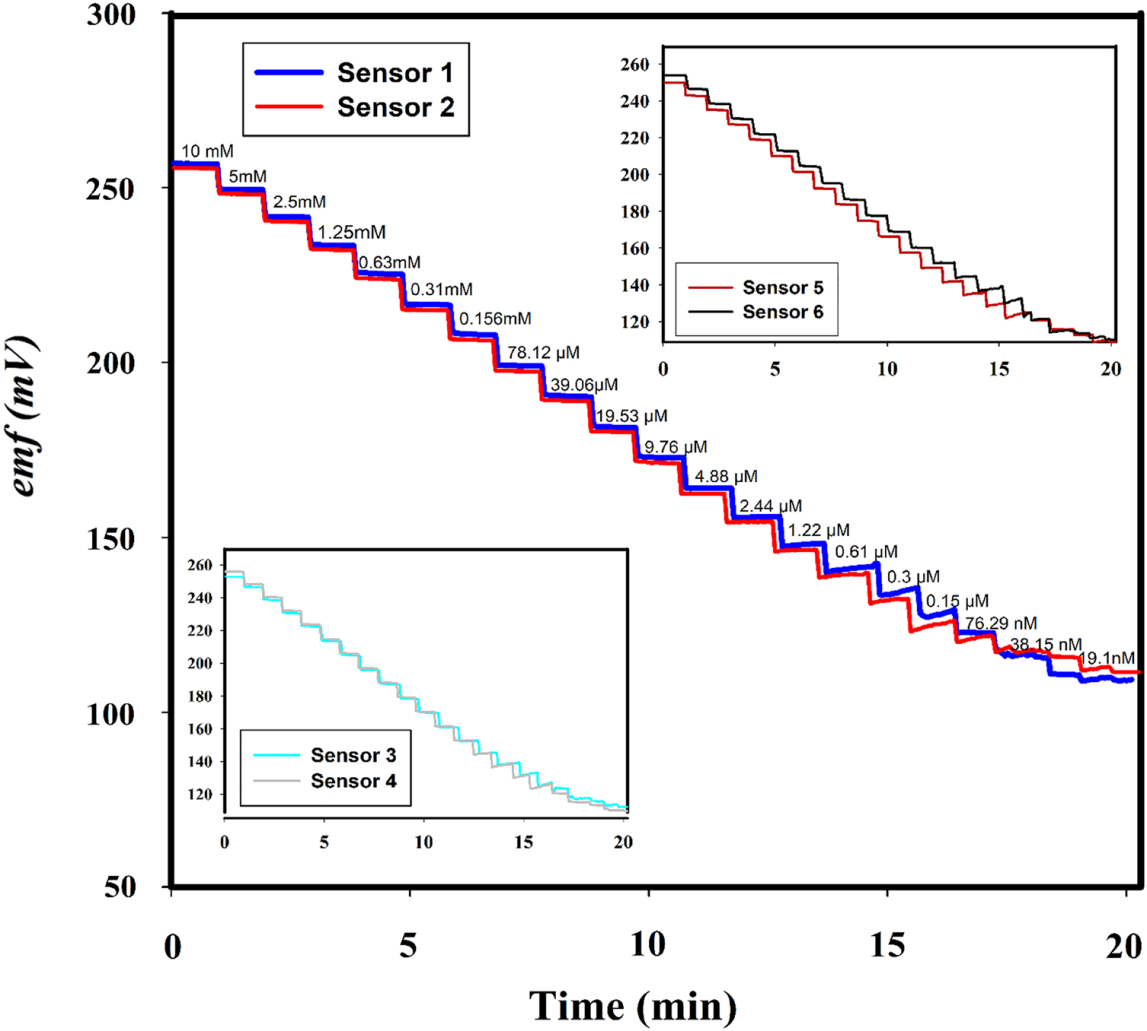
Potential-time curves of six sensors were obtained by measuring the respective emf values in six different concentrated DB solutions (each 10.0 mM) all undergoing successive dilutions in which repeated removal of an aliquot of the respective sample and addition of equivalent volumes of BRB solutions (pH = 7.0). The nearly superimposed profiles of the six different screen-printed sensors highlight the reproducibility of the sensors.

**Figure 4:**
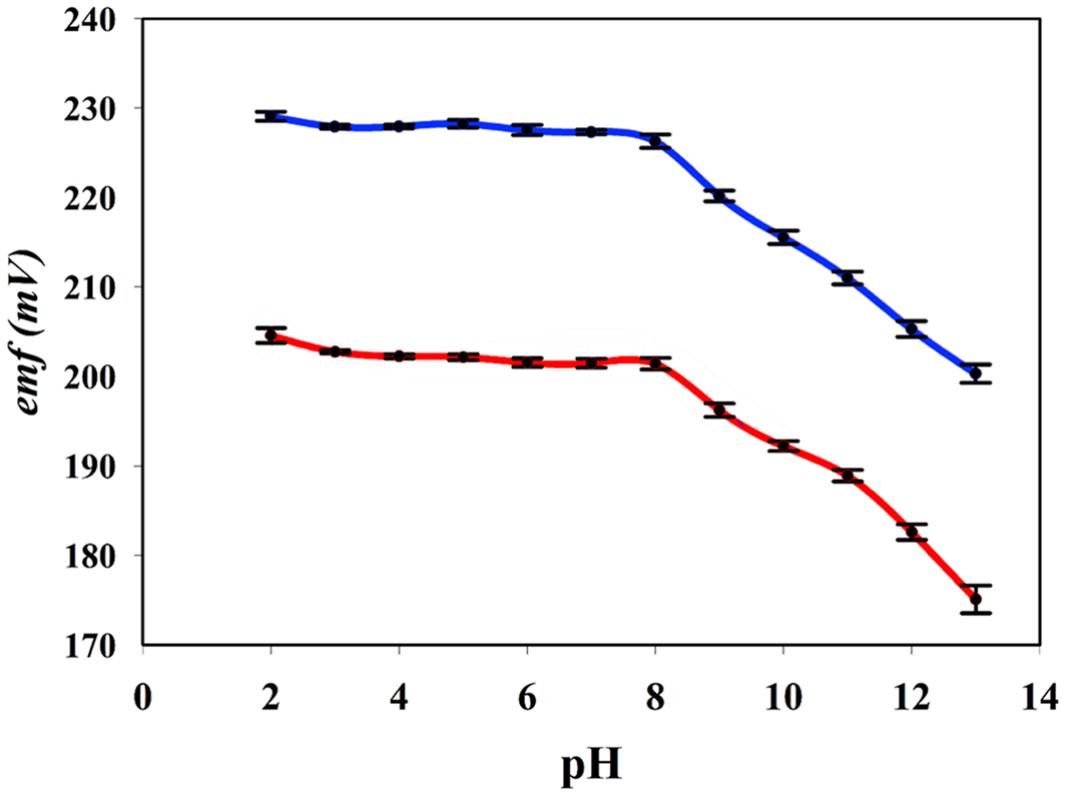
The effect of pH on the potential stability of a DB-sensor using two concentrations of DB; 1.0 and 0.1 mM as shown in the upper blue and lower red lines respectively. Minimal emf changes for pH values between 3 and 8 were observed. Each point is the average of six measurements while the error bars shown represent the RSD values of the six measurements.

The potentiometric time traces obtained by measuring the emf in solutions of DB employing six different screen-printed ion-selective electrode sensors connected to a smartphone (n = 6) are shown in Fig. 3 as plots of the emf values obtained against the logarithmic concentrations of DB with a LOD of 78.0 nM. The average response time for the sensors employed was less than 10 s, which allowed for rapid emf recordings and near real time response during the kinetic monitoring of DB hydrolytic degradation. The measurements were obtained by stepwise dilution of the DB solution, at a 10 mM initial concentration, that was performed by removing an aliquot of the DB solution and its replacement with an equal volume of Britton–Robinson buffer (BRB) at pH 7.0 under continuous emf measurements. A typical calibration curve as a function of DB concentration obtained from the average of the six-potentiometric time traces is presented in Figure S7 in Supplementary Materials. This shows a Nernstian linear dynamic range of several orders of magnitude with a R^2^ = 0.9986 for the linear correlation, an average slope value of 27.51 ± 0.72 mV dec^−1^ and a standard potential, E^0^, of 305.44 ± 1.45 mV. The pH effect on the potentiometric measurements was studied by calibrating the sensor at pH values in the range of 2–13 by using BRB. These results presented in Fig. 4 show no significant difference in the corresponding Nernstian slopes within the pH range 3–8. Figure 4 also shows that at pH values ≥ 9, non-Nernstian slopes were obtained which is attributed to the degradation of the drug.

It was demonstrated with different tests using separate solutions that the sensors exhibited greater selectivity towards DB relative to its degradation product THMP. This may be due to the higher lipophilicity of DB compared to THMP at the working pH, where log *K*^pot^_DB.THMP_ = − 2*.*50. This discrimination towards DB over its degradation product THMP is a factor in allowing accurate monitoring of DB during the initial stages of its chemical catalyzed hydrolysis degradation reaction which were studied by continuous recording of the emf values for DB solutions at pH values 10 and 11 each at thermostatically controlled temperatures of 25, 30 and 35 °C all ± 2 °C and at an initial DB concentration of 0.1 mM.

### *At-line* spectrophotometric measurements

**Figure 5:**
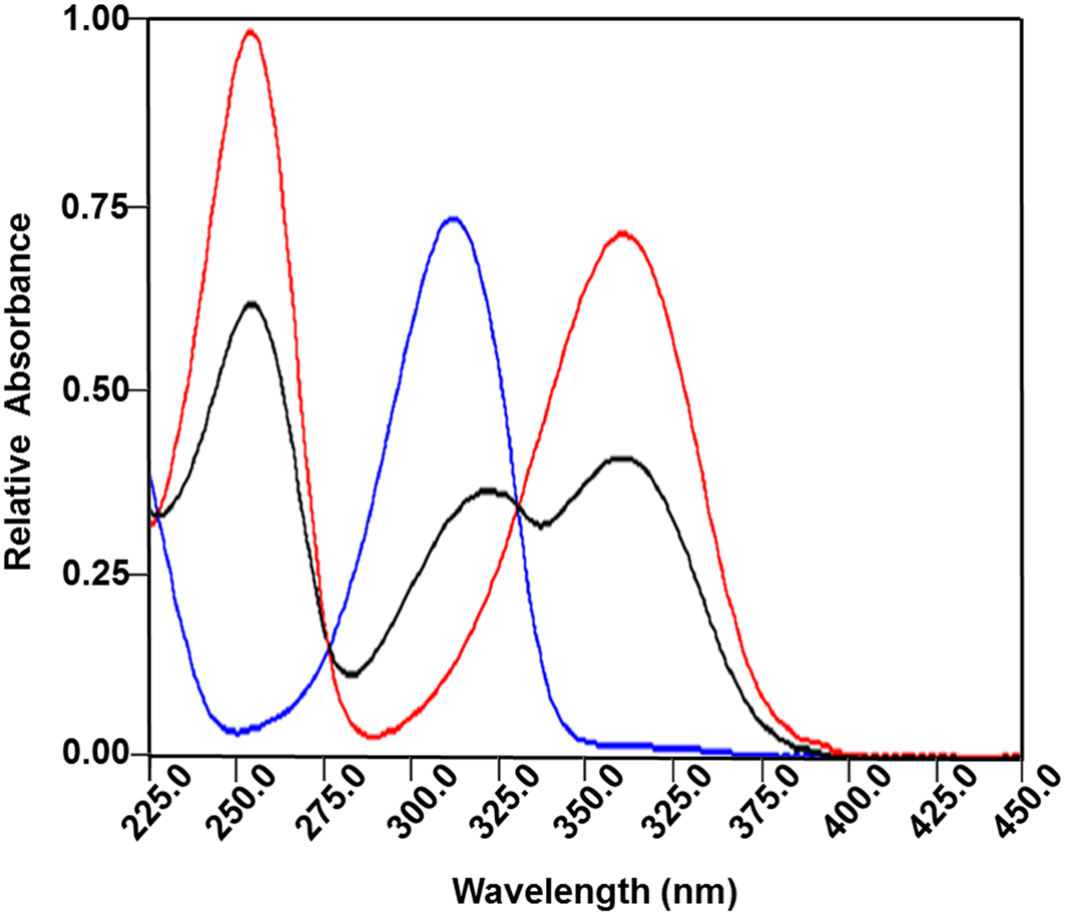
Absorption spectra of 40 μg/mL THMP at pH values of 3.1, 4.2, and 6.1 as represented by the blue, black and red lines respectively.

**Figure 6:**
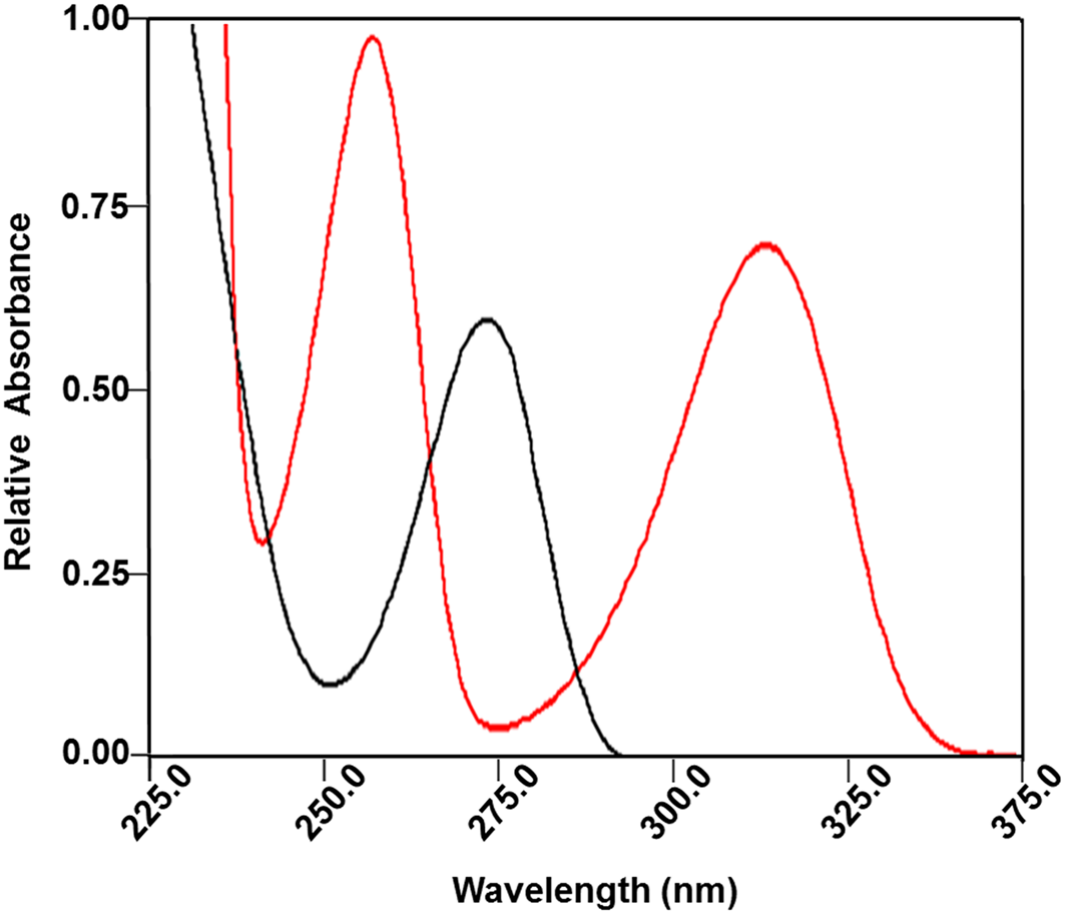
Absorption spectra of 40 μg/mL in each of DB (black line) and its degradation product, THMP (red line) in BRB at pH 7.

DB degrades to give THMP, which in turns undergoes changes in its spectral profile as shown in Fig. 5. These pH dependent spectral profile changes are most likely due to the reversible ionization of the phenolic hydroxyl group conjugated with the pyridine ring. Figure 5 shows the appearance of a THMP absorbance peak at λ_max_ = 320 nm at pH 4 and 6. This is a spectral region where no absorption of the intact drug DB occurs as shown in Fig. 6 where an overlay of DB and THMP spectra both at pH 7 are presented.

**Figure 7:**
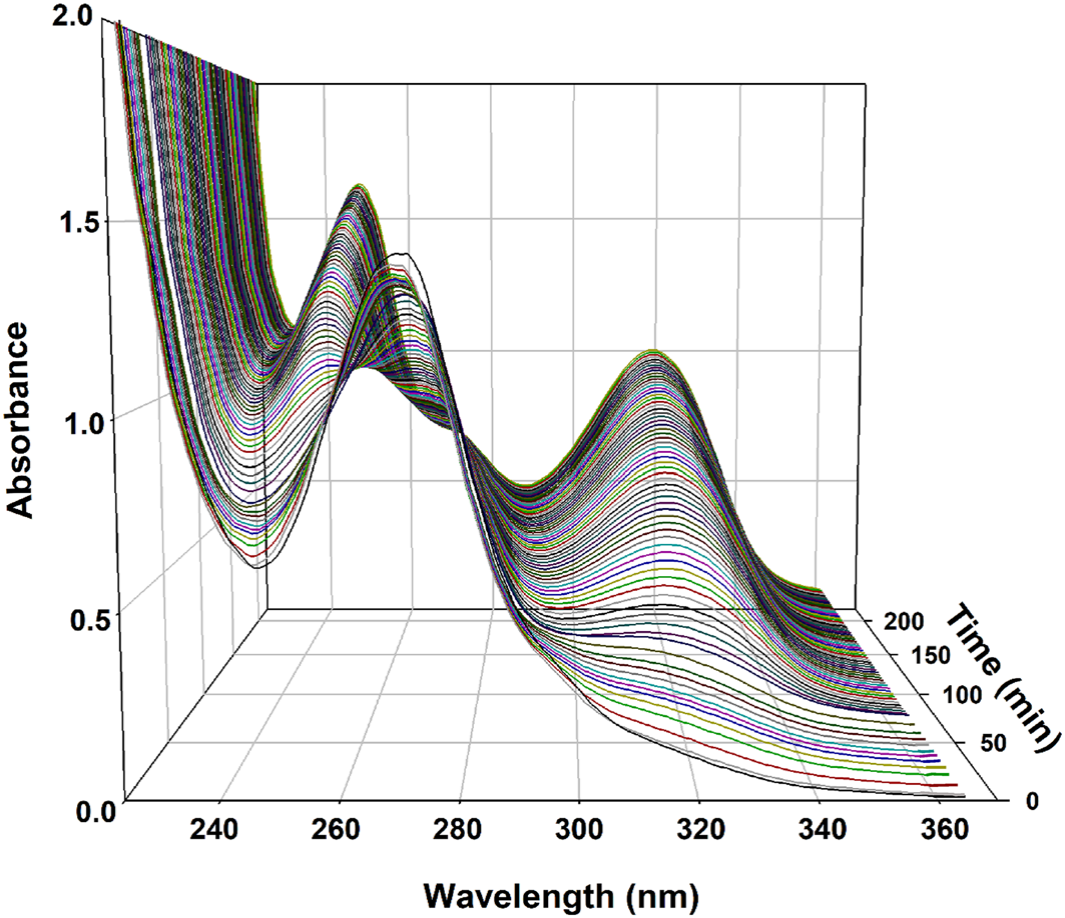
Three dimensional representation of UV absorption spectra of distigmine in alkaline buffer (BRB of pH 10) taken over 3.5 h of hydrolysis at 25 °C, during which the signal due the distigmine observed at 270 nm was gradually reduced and the signals due to the hydrolysis product, THMP, at 252 and 320 nm were gradually increased.

The proposed method depends on measuring the difference between the spectrum of THMP obtained from the hydrolysis of DB and the spectrum of the same concentration of the intact drug. In this way, any signal due to amount of the degradation products present in the DB samples was subtracted from the readings at the corresponding wavelength after hydrolysis and the difference in absorbance before and after hydrolysis would correspond only to the intact drug. Calibration is then obtained by accurately transferring volumes of DB equivalent to 100.0–600.0 μg into a series of test tubes, each containing 1.0 mL 0.05 M NaOH, and were kept in a water bath at 100 °C for 10 min. The contents of each test tube were neutralized using 0.05 M HCl to pH 7.0 and transferred quantitatively in separate 10 mL volumetric flasks which were subsequently completed to the mark with BRB at pH 7. The absorbance of each solution was then recorded at 320 nm against a corresponding blank solution containing the same concentration of intact DB in BRB at pH 7. Figure S8 of the Supplementary Material shows the results of applying this suggested procedure to obtain differences between the spectra at λ = 320 nm for concentrations of 10.0–60.0 μg/mL for the DB degradation product, THMP, against the same respective concentrations of intact DB as blank. This procedure has also produced a strong linear response with a correlation coefficient R^2^ = 0.9999 as shown in Figure S9 of the Supplementary Material. The hydrolytic degradation of DB under the same conditions to produce THMP as previously described above was investigated. Complete hydrolytic degradation of DB to produce THMP was achieved at several concentrations of DB by accurately measuring 100–600 μg volumes of DB which were transferred separately into a series of test tubes. To each test tube, 1.0 mL of 0.05 M NaOH was added then kept in a water bath at 100 °C for 150 min. Separately, the contents of each test tube were transferred quantitatively into six 10 mL volumetric flasks which were subsequently completed to the mark with BRB. In each sample THMP absorbance signal was recorded at 320 nm, as this wavelength minimized interfering signal from DB as shown in Fig. 6, against the corresponding blank solution containing the same concentration of intact DB dissolved in BRB at pH 7 allowed for the construction of a THMP calibration curve. DB degradation in two BRB solutions of pH 10 and 11 thermostatically controlled at 25, 30 and 35 °C all ± 2 °C were investigated. This was accomplished by scanning the DB degradation reaction every 3 min from the start of the reaction over the 150 min time span at the respective controlled temperatures through the increase of the THMP signal at 320 nm that also corresponds to the decrease of DB signal at 270 nm with increasing time as shown in Fig. 7.

### *Off-line* chromatographic measurements

**Figure 8:**
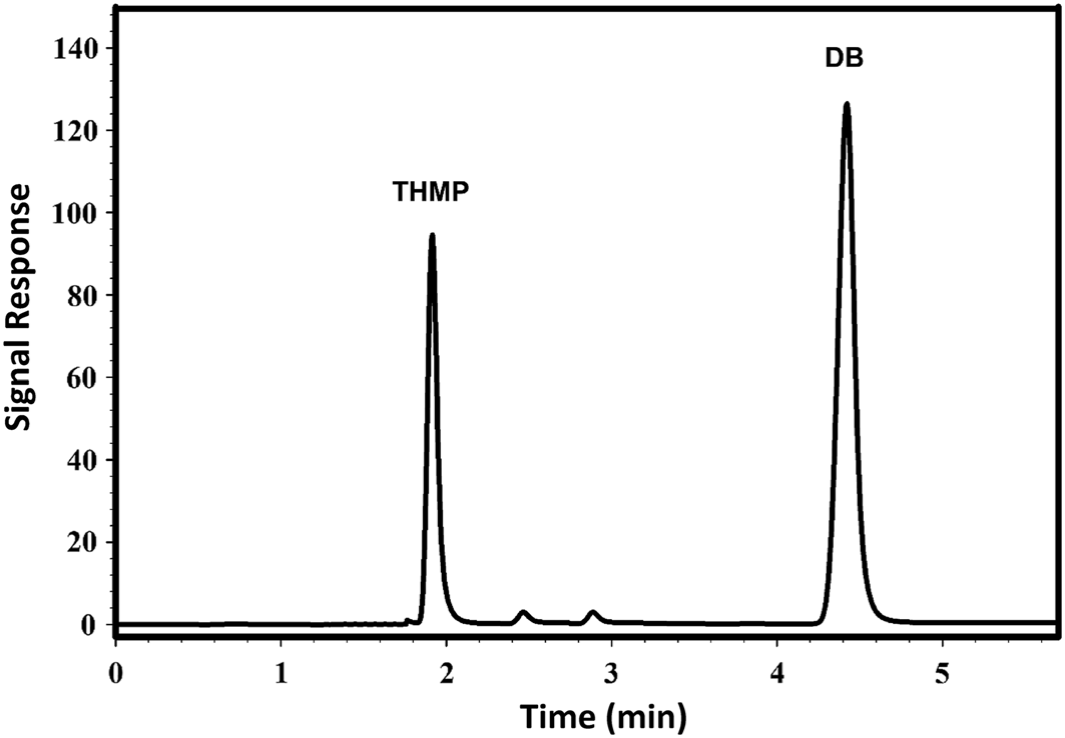
HPLC chromatogram of distigmine, (T_R_ = 4.45 min) and its degradation product, THMP, (T_R_ = 1.91 min), on a C_18_ Zorbax TM analytical column, using aqueous 0.01 M sodium-1-heptane sulphonate (adjusted to pH: 3.0 with dilute phosphoric acid): acetonitrile (70:30 v/v) as a mobile phase with 1.0 mL/min flow rate and UV detection at 220 nm.

In HPLC measurements, sodium-1-heptane sulphonate was used as an ion-pairing agent in order to allow for the complete separation between DB and its THMP and to improve peak symmetry and sharpness as well as reduce peak tailing of the drug. The amount of acetonitrile used was also adjusted, since at higher acetonitrile concentration, separation occurred but with excessive tailing and increased retention time of the DB peak. Using a 0.01 M sodium-1-heptane sulphonate:acetonitrile (70:30 v/v) mobile phase and the chromatographic conditions previously described, a retention time of 4.45 ± 0.02 min for DB and 1.91 ± 0.02 min for its alkaline degradation product THMP was achieved with excellent peak shapes as shown in Fig. 8. Using the external standard approach for calibration, a linear correlation R^2^ = 0.9996 was obtained between the relative peak area and the corresponding drug concentrations in the range 4–32 µg/mL at λ = 220 nm as shown in Figure S10 of the Supplementary Material.

**Figure 9:**
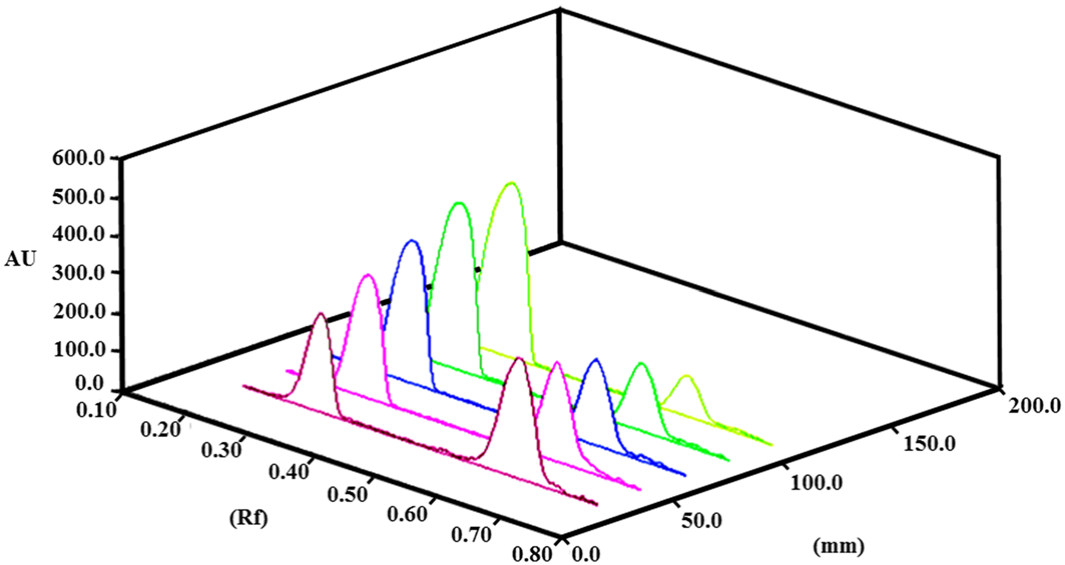
3D densitometric scanning profiles for TLC-chromatograms of different concentrations of distigmine bromide (2- 12 μg/spot) at 270 nm showing DB and THMP peaks at Rf values of 0.31 and 0.64, respectively.

For TLC densitometry, many experiments were conducted to achieve optimum separation of the drug and its degradation product. The trials were focused on changing the composition of the developing system by varying the percentages of organic solvents. These systems included methanol : ethyl acetate : NH_3_ (4:1:0.2, by volume); acetone:butanone:H_2_O:NH_3_ (5:3:2:0.01, by volume) and acetone:butanone:acetic acid:H_2_O:NH_3_ (1:1:1:1:0.03, by volume). The first developing system showed poor resolution for the two analytes with peak tailing. The second system was not suitable for DB, which remained almost on the spotting line. However, upon adding acetic acid to the developing system, the obtained TLC chromatogram was promising. After fine adjustment of the ratios and addition of acetic acid, the optimum developing system was acetone:butanone:acetic acid:H_2_O:NH_3_ (1:1:1:1:0.03, by volume). Figure 9 shows sharp symmetric peaks with good resolution for the two components with Rf values 0.31 and 0.64 for DB and THMP, respectively. This separation allowed for the scanning of DB at the corresponding wavelength without any interference from its degradation product. A linear correlation, R^2^ = 0.9996, was determined between the integrated area at 270 nm and the corresponding concentration of drug in the range of 2.0–12.0 μg/spot as shown in Figure S11 of the Supplementary Material.

*Off-line* monitoring of the hydrolytic degradation of DB using HPLC and TLC were performed. In both cases an aliquot of the DB degradation reaction was removed every 3 min from the start of the reaction and for 100 min. These aliquots were neutralized to pH 7 with BRB and 20 µL were injected in the HPLC using the same chromatographic conditions employed for the assay measurements of DB as described earlier. These conditions produced complete separation and excellent peak shapes for DB and THMP, whose retention times were verified by separate injections of each of these compounds under the same chromatographic conditions. In the case of TLC, the aliquots removed from the DB degradation reaction, were spotted after their neutralization onto silica gel 60 F_254_ TLC plate employing the same TLC chromatographic conditions previously described. The intensities of the peaks of the analyte of interest were measured and relative concentration profiles were determined from the relative peak areas versus reaction times.

### Validation of developed methods

All the methods employed in this work were validated for accuracy, precision, specificity, repeatability, linearity, range, detection and quantification limits according to the ICH guidelines^46^ and applied to the analysis of laboratory prepared mixtures containing different ratios of DB and THMP. The accuracy of the obtained ISE, UV, HPLC and TLC results expressed as the average of three measurements for each of three concentrations of DB 1.0 × 10^–3^, 1.0 × 10^–4^ and 1.0 × 10^–5^ M; 15.0, 35.0 and 55.0 µg/mL; 10.0, 22.0 and 30.0 µg/mL; and 5.0, 7.0 and 9.0 µg/spot respectively are presented in Table 1. The intra-day repeatability and the inter-day intermediate precision of the each technique as expressed in each case as the RSD % of three measurements for each of three concentrations of DB being 5.0 × 10^–3^, 6.0 × 10^–4^ and 8.0 × 10^–5^ M; 20.0, 30.0 and 50.0 µg/mL; 8.0, 16.0 and 24.0 µg/mL and 2.0, 6.0 and 10.0 µg/spot for ISE, UV, HPLC and TLC respectively are also presented in Table 1 which also summarizes several metrological parameters of the proposed sensor showing the suitability of all of the developed methods for their proposed use as evaluated with respect to IUPAC recommendations^47^. Table 1 also shows that the proposed ISE method possesses one of the widest linear ranges and the lowest LOD and LOQ values of all the methods employed here. The proposed methods were also employed successfully for assaying DB in Ubretid® tablets. The reliability and accuracy of recoveries of all the proposed methods as listed in Table 2 relative to the official method for DB assay clearly show that the proposed methods are unaffected by excipients and other non-active components present in commercial formulations.

**Table 1:** Metrological parameters (regression and validation data) of the techniques employed for distigmine determination.

Parameter	ISE	UV	HPLC	TLC
Accuracy (mean ± S.D.)^a^	100.51 ± 0.531	100.53 ± 0.482	100.19 ± 1.344	100.62 ± 0.791
Specificity	100.49 ± 1.486	100.78 ± 0.662	99.93 ± 1.424	100.42 ± 1.686
Repeatability^b^	0.671	0.971	1.197	0.783
Intermediate precision^c^	0.771	1.087	1.234	0.962
Slope^d^	27.51	0.0196	0.0619	0.2231
Intercept	309.6	0.0095	0.06	0.0101
Correlation coefficient	0.9993	0.9999	0.9998	0.9998
Range	0.15 μM–10.0 mM	17.35–104.10 μM	6.94–55.52 μM	2–12 μg/spot
LOD^e^	0.078 μM	5.22 μM	1.65 μM	0.62 μg/spot
LOQ^f^	0.099 μM	15.82 μM	5.01 μM	1.88 μg/spot

^a^The accuracy (n = 3), average of three concentrations (1.0 × 10^–3^, 1.0 × 10^–4^ and 1.0 × 10^–5^ M for ISE), (15.0, 35.0 and 55.0 µg/mL for UV), (10.0, 22.0 and 30.0 µg/mL for HPLC) and (5.0, 7.0 and 9.0 µg/spot for TLC).

^b^The intra-day (n = 3), RSD% of concentrations (5.0 × 10^–3^, 6.0 × 10^–4^ and 8.0 × 10^–5^ M for ISE), (20.0, 30.0 and 50.0 µg/mL for UV), (8.0, 16.0 and 24.0 µg/mL for HPLC) and (2.0, 6.0 and 10.0 µg/spot for TLC).

^c^The inter-day (n = 3), RSD% of concentrations (5.0 × 10^–3^, 6.0 × 10^–4^ and 8.0 × 10^–5^ M for ISE), (20.0, 30.0 and 50.0 µg/mL for UV), (8.0, 16.0 and 24.0 µg/mL for HPLC) and (2.0, 6.0 and 10.0 µg/spot for TLC).

^d^Average of three determinations.

^e^Limit of detection for ISE is measured according to IUPAC by interception of the extrapolated arms of nonresponsive and Nernstian segments of the calibration plot of Figure S7. While for other methods, LOD is calculated according to ICH from the standard deviation (σ) of the regression residuals and the slope of the calibration curve (S) according to the following equations: LOD = 3.3(σ/S).

^f^LOQ is calculated according to ICH from the standard deviation (σ) of the regression residuals and the slope of the calibration curve (S) according to the following equations: LOQ = 10(σ/S).

**Table 2:** Determination of distigmine in pharmaceutical formulation using the developed methods and the official method.

Pharmaceutical formulation	Recovery (%) ± S.D.^a^				
	ISE	UV	HPLC	TLC	Official method^b^
Ubretid**®** tablets (5 mg DB/tablet)	100.25 ± 0.507	101.02 ± 0.661	99.94 ± 1.260	98.21 ± 1.405	99.43 ± 0.788
Student’s t-Test^c^ (2.776)	1.516	2.678	0.594	1.312	
F value^c^ (19.00)	2.416	1.421	2.557	3.179	

^a^Average of three determinations.

^b^Japanese pharmacopeia 2001 “Direct spectrophotometric measurement at 270 nm in 0.1 M hydrochloric acid”.

^c^The values in parentheses are the corresponding theoretical values for t and F at *P* = 0.05.

### Degradation kinetics

The analytical methods employed here are all stability indicating and were employed for monitoring the degradation kinetics of DB at pH 10 and 11 in each case at 25, 30 and 35 °C all ± 2 °C. For *in-line* ISE measurements, the rapid response time for DB of less than 10 s offered multiple advantages. This allowed for near instantaneous emf recording and thus near real time observation of the hydrolysis behaviour of DB. This also allowed for a significantly larger number of data points to be obtained relative to the *at-line* and *off-line* methods employed here which increases the confidence in constants determined for the kinetics of DB hydrolysis based on data obtained through this *in-line* approach. The near instantaneous response time of the sensor also facilitated the recording of the emf at pH 10 and 11 before the start of the DB hydrolysis process. This in turn allowed for calibration curves obtained at these pH values employing this methodology to show no statistically significant differences in their linearity or Nernstian slopes relative to those obtained by the same method at pH 7.

*At-line* spectrophotometric measurements were conducted to investigate DB degradation at the same pH and temperature conditions mentioned above. Here, complete hydrolytic degradation of DB to produce THMP was achieved at several concentrations of DB by accurately measuring volumes of DB, equivalent to 100–600 μg, which were transferred separately into a series of test tubes. To each test tube, 1.0 mL of 0.05 M NaOH was added then kept in a water bath at 100 °C for 150 min. Separately, the contents of each test tube were neutralized using 0.05 M HCl to pH 7.0 and transferred quantitatively into six 10 mL volumetric flasks which were subsequently completed to the mark with BRB (pH 7). In each sample THMP absorbance signal was recorded at 320 nm, as this wavelength eliminated interfering signal from DB against the corresponding blank solution containing the same concentration of intact DB dissolved in BRB at pH 7 allowing for the construction of a calibration curve.

The DB hydrolytic degradation studies were performed by auto scanning the reaction every 3 min over a period of 3.5 h. These conventional scans are shown in Fig. 7 where the DB peak at 270 nm gradually decreased and the THMP peaks at 252 and 320 nm increased over time. Figure 7 and more visibly in Movie S12 of the Supplementary Material show two isosbestic points at 260 and 287 nm that indicate the absence of side reactions. The kinetic degradation of DB can thus be expressed either directly through the decrease of DB signal at 270 nm or through the increase of THMP signal at 320 nm with time. Both these approaches consistently correlated fairly well with that obtained by ISE.

*Off-line* experiments were performed for monitoring the degradation of DB by placing a fixed concentration in BRB at pH 10.0 and 11 all at 25 ± 2 °C where aliquots were withdrawn at 5 min intervals. These aliquots were neutralized to pH 7 and completed to a definite volume, and subsequently spotted on the silica gel 60 F_254_ TLC plates and injected in the HPLC using the chromatographic conditions described previously for each technique. For both methods, relative decreasing concentration profiles of DB were determined versus reaction times.

**Figure 10:**
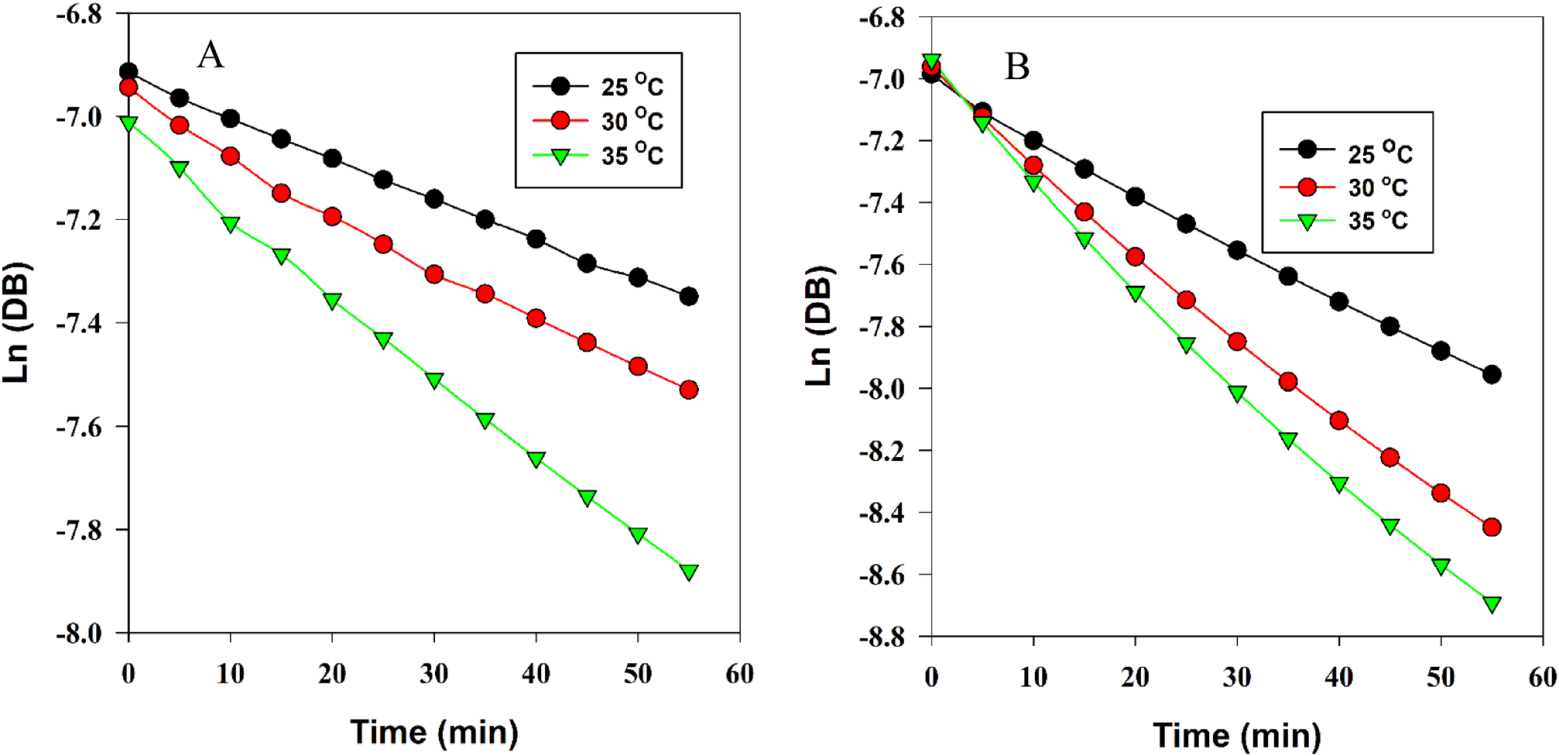
Pseudo-first-order kinetics hydrolysis plots of 1.0 mM distigmine bromide solutions with BRB of pH 10 (Panel **A**) and 11 (Panel **B**) at three different temperatures as obtained by a smart phone integrated potentiometric sensor.

For all of the approaches employed in this work the logarithm of the DB concentration was then plotted with respect to time for measurements at pH 10 and 11 at each of the three temperatures employed as shown in Fig. 10 in the case of the smart phone integrated potentiometric sensor. These plots all show pseudo-first-order degradation of DB through its hydrolysis in a large excess of BRB. From these plots, degradation rate constants and the corresponding half-lives of DB, t_1/2_ were determined and are all shown to be in good agreement across all methods employed as summarized in Table 3. This data also shows the hydrolysis rate of DB increasing and its half-life, t_1/2_, decreasing with increasing temperature and pH. The activation energy, E_a_, for the degradation reaction was calculated using Eq. 1^48^.

1 $$\log \frac{k2}{{k1}} = \frac{Ea}{{2.303\;R}}\left( {\frac{T2 - T1}{{T1\;T2}}} \right)$$

**Table 3:** Kinetic data of distigmine alkaline degradation monitored by the developed methods.

Developed method	pH of buffer	Temperature (°C)	K (min^−1^)	T_1/2_ (min)	T_90%_ (min)
ISE	10	25.0	0.0075	92.40	13.73
		30.0	0.0096	72.188	10.73
		35.0	0.0124	55.88	8.31
	11	25.0	0.0181	38.29	5.69
		30.0	0.0240	28.88	4.29
		35.0	0.0324	21.39	3.17
Spectrophotometry	10	25.0	0.0077	90.00	13.37
		30.0	0.0097	71.44	10.62
		35.0	0.0130	53.31	7.92
	11	25.0	0.0177	39.15	5.82
		30.0	0.0252	27.50	4.09
		35.0	0.0338	20.51	3.18
HPLC	10	25.0	0.0081	85.56	12.72
		30.0	0.0110	63.00	9.36
		35.0	0.0137	50.58	7.52
	11	25.0	0.0195	35.54	5.28
		30.0	0.0277	25.02	3.72
		35.0	0.0346	19.97	3.18

These activation energies were 9.90 ± 0.97, 10.67 ± 1.80 and 10.02 ± 2.15 kcal mol^−1^ as obtained by ISE, spectrophotometry and HPLC respectively, which coincides with the reported values for the hydrolytically susceptible ester group^49^.

### Evaluation of the greenness profile of the proposed methods

In this work, all proposed methods were evaluated and ranked in light of their adherence to green analytical chemistry principles^1^ according to GAPI^40^, ESA^41^ and AGREE^42^. The GAPI assessment tool serves as a valuable semi-quantitative technique for assessing the overall greenness of an analytical technique. This approach expands on previous assessment tools through rating analytical methods on various aspects including sample preparation, collection, safety, potential health impact of the substances used as well as waste produced. GAPI uses a three-color scale for each sector, being green to yellow to red, similar to traffic lights, where green denotes a benign technique and red denotes a non-environmentally friendly one while yellow is somewhere in the middle. The GAPI pictograms obtained upon employing this system on all our proposed techniques, as shown in Table 4, demonstrates that the smart phone integrated ISE method was the greenest approach with green in 13 sectors, while the spectrophotometric, HPLC and TLC methods had six, three and four sectors green respectively.

**Table 4:** Comparison of the greenness profiles of proposed methods using GAPI, ESA and AGREE tools.

Method	Greenness profile assessment tool		
	GAPI	ESA	AGREE
ISE		94	
Spectrophotometry		91	
HPLC		86	
TLC		70	

The ESA was applied for a more comprehensive assessment of the greenness profile of the proposed methods. This scale assigns penalty points to each non-green procedural component, which are then subtracted from a base of 100 to provide a final numerical score. The higher the score the greener is the analytical process. Table 4 lists these scores, as computed for the proposed methods in this work, showing the smartphone based electrochemical sensor approach to be the most green followed by spectrophotometry, HPLC and TLC. This scale has the advantage of being semi-quantitative, with numerical values as a reference for assessing greenness. However, there are also many disadvantages to using this scale. These include not providing information about the type of hazards or the factors that contribute to the negative environmental impact of an analytical procedure, all information that is most useful in the development of a better eco-friendly method.

For this reason a third approach, AGREE, which is an all-encompassing, straightforward and flexible assessment tool that provides interpretable and instructive results, that was recently introduced, was employed here^47^. In this approach, the assessment criteria were derived from the 12 principles of GAC. The tool output is a clock-like graph, with color representation similar to the GAPI system but with an overall score displayed in the middle. One of the advantage of this metric is the availability of open-source, downloadable software (https://mostwiedzy.pl/AGREE), which simplifies its application. This assessment tool also indicates that the ISE methodology employed in this work possessed the highest score, 0.88, and highest number of green segments, followed by UV–VIS, HPLC and TLC with scores of 0.68, 0.51 and 0.45 respectively as listed in Table 4.

In addition to its greenness profile, the smartphone-based ISE technique offers several unique advantages. These include its fast response time providing the ability for continuous real time reaction monitoring as discussed earlier; its compatibility with microfabrication processes; its portability and miniaturization permitting its use for in-situ measurements; its affordability and ease of use; its high reproducibility at a wide linear range as well as its nano-molar level LOD. These unique advantages suggest the suitability of this method for in vitro kinetic testing in possible conjunction with ingestible electronics.

## Conclusion

Four novel stability-indicating methods for DB were reported and tested on commercial formulations. This represents a major advancement for the analysis of DB for which no stability-indicating method was previously reported. The methods described here were an *in-line* method based on a smartphone connected to a screen-printed selective electrode hand-held device, an *at-line* UV-spectrophotometric strategy and two *off-line* strategies, utilizing HPLC and TLC methods. All the methods were validated for accuracy, precision, specificity, repeatability, linearity, range, detection and quantification limits according to the ICH guidelines and were applied to the analysis of laboratory prepared mixtures as well as formulated commercial products. The analytical methods employed here were applied for monitoring the degradation kinetics of DB at pH 10 and 11 in each case at 25, 30 and 35 °C all ± 2 °C. For all of the methods of pharmaceutical analysis used and for all measurement conditions, the logarithms of the DB concentrations were plotted with respect to time. All plots showed pseudo-first-order degradation of DB through its hydrolysis in a large excess of BRB. From these plots, degradation rate constants and the corresponding half-lives of DB, t_1/2_ were determined and are all shown to be in good agreement across all methods employed. The activation energy for the degradation reaction was estimated to be 9.90 ± 0.97, 10.67 ± 1.80 and 10.02 ± 2.15 kcal mol^−1^ as obtained by ISE, spectrophotometry and HPLC respectively. While all stability-indicating methods proposed were shown to be highly reliable, the smartphone integrated selective sensor is highlighted as a portable analyzer with potential high cloud connectivity and was shown to combine a wide linear dynamic range, the fastest response time with the lowest limits of detection and quantitation while best integrating green analytical chemistry principles.

## Methods

### Chemicals and reagents

Potassium tetrakis 4-chlorophenyl borate (KTCPB), calix-6-arene (CX6), Polyvinyl chloride (PVC), 2-nitrophenyl octyl ether (NPOE), 2-methyltetrahydrofuran (2-MeTHF), tetrahydrofuran (THF), acetone, acetonitrile and ammonia solution 32% were purchased from Sigma-Aldrich (St. Louis, MO, USA). Acetic acid, butanone, sodium-1-heptane sulphonate, boric acid, phosphoric acid and sodium hydroxide were purchased from Merck (Gernsheim, Germany). Distigmine bromide (DB) reference standard and Ubretid® tablets (5 mg DB/tablet) batch numbers 510123, 503600 and 5406132 were kindly supplied by The Arab Drug Company (Cairo, Egypt). Screen-printed three electrode strips C110, were purchased from Drop Sens, Metrohm (Spain).

### Analytical methodology

#### *In-line* smartphone-based ISE measurements

A screen-printed DB-selective membrane composed of PVC (33.50%), NPOE (65.71%), KTCPB (0.16%, 5.0 mmol kg^−1^) and CX6 (0.63%, 10.0 mmol kg^−1^) were dissolved in 2-MeTHF THF (6.0 mL). The sensing membrane solution was directly applied on the SPE by the aid of a micropipette and allowed to dry out overnight. The sensor was subsequently preconditioned by soaking it in a 0.1 mM solution of DB for 3.5 h at room temperature before its initial use. Lab-free *in-line* potentiometric measurements were carried out by a hand-held, computerized battery-powered PalmSens4 potentiostat/galvanostat (Palm Instruments BV, The Netherlands) interfaced with a smart phone controlled by PStouch mobile app. The screen-printed C110 strip employed consisted of three 4 mm diameter electrodes comprising a working carbon electrode, an auxiliary electrode and Ag/AgCl reference electrode. Potentiometric measurements were carried out with the three-electrode system in contact with the test solution. Calibration curves for DB-selective electrodes were obtained by the successive dilution method, starting with a 10.0 mM DB solution and using buffer solution of pH 7.0 as the diluent, under continuous measurements of the electromotive force. Calibration curves were constructed by plotting the emf values versus the logarithm of DB concentrations. The performance of the electrodes were validated in accordance with the IUPAC recommendation^47^.

#### *At-line* spectrophotometric measurements

Spectrophotometric *at-line* absorption measurements employed a UV–Vis double beam PC 8 scanning auto cell spectrophotometer, UVD 3200 (Labomed, INC, USA). DB determination was achieved by measuring the difference between the spectrum of the alkaline degradation product and the spectrum of the same concentration of the intact drug.

#### *Off-line* chromatographic measurements

The first *off-line* technique employed in this work made use of an Agilent HPLC system equipped with a G1310A isocratic pump, a G1314 variable wavelength detector and a Rheodyne injector (model 7725I). The HPLC measurements were performed on 20 µL samples using a 5 μm C_18_ Zorbax TM analytical column (25 cm × 0.46 cm) and a mobile phase of 0.01 M sodium-1-heptane sulphonate : acetonitrile (70:30 v/v) at a flow rate of 1.0 mL min^−1^ and 220 nm UV-detection. The sample and the mobile phase were filtered by 0.22 μm and 0.45 μm Millipore membrane filters, respectively. The mobile phase was then degassed in an ultrasonic bath for 15.0 min immediately before use. Aliquots of DB equivalent to 40.0–320.0 μg were accurately transferred into 10 mL volumetric flasks and the volume was completed with mobile phase. Calibration curves were then constructed for DB by plotting the relative peak areas of DB as a function of respective concentrations.

The second *off-line* technique selected in this work was densitometry. The device employed was equipped with a UV lamp, a Camag Linomat 5 autosampler with a 100 µL micro-syringe, a TLC scanner-Model 3 S/N 130319 and winCats software for densitometric evaluation (CAMAG, Muttenz, Switzerland). The measurements were obtained using 20 cm × 20 cm TLC plates pre-coated with a 0.25 mm thick layer of silica gel 60 F_254_ (E. Merck, Darmstadt, Germany). The solutions examined were applied as separate spots 20 mm from the bottom of the plates each with a 2 mm band length and developed at 25 ± 2 °C in the absorbance mode at 270 nm running a developing system of acetone:butanone:acetic acid:H_2_O:NH_3_ (1:1:1:1:0.03 by volume). The drug solutions examined were applied as separate compact spots 15 mm from the bottom of the plates each with a 3 mm band length which were placed in a chromatographic tank saturated with the mobile phase for 30 min prior to development. The normal phase TLC-plates were developed over 8 cm in an ascending manner then left to dry in air and then were scanned at 220 nm. In order to calibrate this method, accurately measured aliquots equivalent to 2.0–12.0 μg DB were spotted on the TLC plates, using the Camag Linomat autosampler using the same TLC chromatographic conditions as stated above. Calibration graphs relating the optical density of each spot to the corresponding concentration of DB were constructed.

### Computational details

Molecular orbital calculations for CX6, distigmine in its non-brominated form (DG) and their host–guest complexes were performed in the gas phase at the DFT level of theory using the Gaussian16 code^50^. All geometries were optimized without any constraints using the B97D exchange and correlation functional with the 6-31G(d,p) basis set for all atoms except for oxygen, for which the 6-31 + G(d,p) basis set which includes a diffuse function was employed. A similar computational protocol was reported to be successful for similar systems^18,51^. All critical points were characterized as local minima by means of harmonic vibrational analysis at the same level of theory by ensuring the absence of negative or imaginary frequencies. These frequency calculations were also used to calculate the Gibbs free energies for all species calculated. To account for solvent effects, the implicit polarizable continuum model (PCM)^52^ was employed where a single point calculation using the gas phase optimized geometries were computed with the standard Pople type 6-311 + + g(d,p) basis set for all the atoms to calculate the solvation free energies. Since the medium employed experimentally, 2-nitro phenyl octyl ether, having a dielectric constant ε ¼ 23–24^53–56^ is unavailable as a solvent in the Gaussian code, the solvent 1-nitro propane, having a dielectric constant of ε ¼ 23.7 was employed in these PCM calculations. The Gibbs free energies in solution were calculated for CX6, DG and complex between them as the sum of the gas-phase free energy ${\Delta G^\circ }_{gas}$ and the solvation free energy ${\Delta G^\circ }_{solv}$, as previously reported^18,20,51^. The binding Gibbs free energy is then calculated using ${\Delta G^\circ }_{solv}$ values as

2 $$\Delta G^\circ_{binding} = \Delta G^\circ_{CXDG} - \left( {\Delta G^\circ_{CX} + \Delta G^\circ_{DG} } \right)$$

where ${\Delta G^\circ }_{CXDG}$, ${\Delta G^\circ }_{CX}$, ${\Delta G^\circ }_{DG}$ are the ${\Delta G^\circ }_{solv}$ of the CX6–DG complex, CX6 and DG respectively. To examine the hydrophobicity/hydrophilicity of the CX6–DG complex relative to each of CX6 and DG we have calculated the log*P* values per the protocol suggested by Nedyalkova et al.^57^. Briefly, full geometric optimization without constrains were performed for all systems under study using the SMD model employing the 6-31G(d,p) basis set for all atoms except for oxygen where the 6-31 + G9d,p) basis set was used. The SMD model was shown to be suitably applied, as a solvation model, to charged and neutral species alike^58^. In this model the solvation free energy is divided into bulk electrostatic and cavity dispersion contributions. The octanol/water partition coefficient, was obtained by using the SMD free energies obtained in these two solvents at 289.15 K which were applied to calculate the standard free energy associated with the transfer of the solute from water to octanol, $\Delta G^\circ_{O/W}$, as shown below;

3 $$\Delta G^\circ_{{{\raise0.7ex\hbox{$O$} \!\mathord{\left/ {\vphantom {O W}}\right.\kern-0pt} \!\lower0.7ex\hbox{$W$}}}} = \Delta G^\circ_{O} - \Delta G^\circ_{W}$$

The octanol/water partition coefficient was subsequently calculated according to

4 $$logP = - \frac{{\Delta G^\circ_{O/W} }}{2.303 RT}$$

The noncovalent interactions (NCI) between DG and CX6 in their complex geometries were calculated by means of reduced density gradients. These gradients were analysed using the Multiwfn software for the generation of an NCI surface where the reduced density gradients (RDG) were plotted using the VMD software employing an isosurface value of 0.3. Subsequently, plots of RDG and sign(λ_2_)ρ values with a RDG and density cutoff of 2.0 and 0.05 a.u respectively were produced to inform about the nature and strength of the NCIs. Molecular electrostatic potential surfaces were created using cuebgen utility in the Gaussian 16 package.

## Supplementary Information

Supplementary Figure 1.

Supplementary Video 1.

## Data Availability

All data generated or analysed during this study are included in this published article and its supplementary information files.
