# Supplementary material for: A green compliant hand-held selective electrode device for monitoring active pharmaceuticals and the kinetics of their degradation
Source: Sci Rep. 2023 Jul 21;13:11792. doi: 10.1038/s41598-023-38416-y (PMC10361986; doi:10.1038/s41598-023-38416-y)
Supplement: Supplementary file 1 — Supplementary Figure 1. [file 41598_2023_38416_MOESM1_ESM.docx]

**Electronic Supplementary Information**

**A green compliant hand-held selective electrode device for monitoring active pharmaceuticals and the kinetics of their degradation.**

**Norhan Badr ElDin^a^, Eslam Dabbish^b^, Esraa Fawaz^a^, Mohamed K. Abd El-Rahman^a,c*^, Tamer Shoeib^b ‡*^**

^a^ Analytical Chemistry Department, Faculty of Pharmacy, Cairo University, Kasr-El Aini Street, Cairo, Egypt 11562.

^b^ Department of Chemistry, The American University in Cairo, New Cairo 11835, Egypt.

^c^ Department of Chemistry and Chemical Biology, Harvard University, 12 Oxford Street, Cambridge, MA 02138, USA

*****Corresponding author e-mail: T.Shoeib@aucegypt.edu (T. Shoeib).

*Corresponding author e-mail: mohamed.khaled@pharma.cu.edu.eg


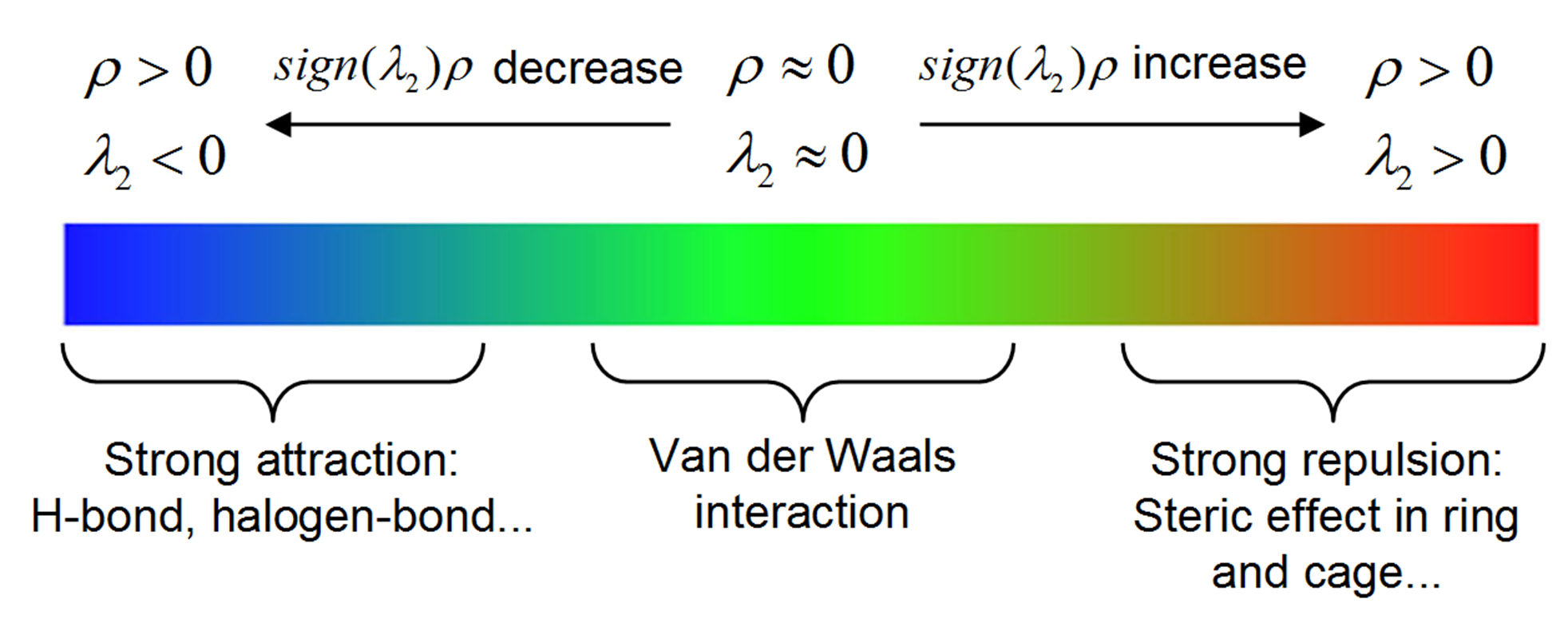

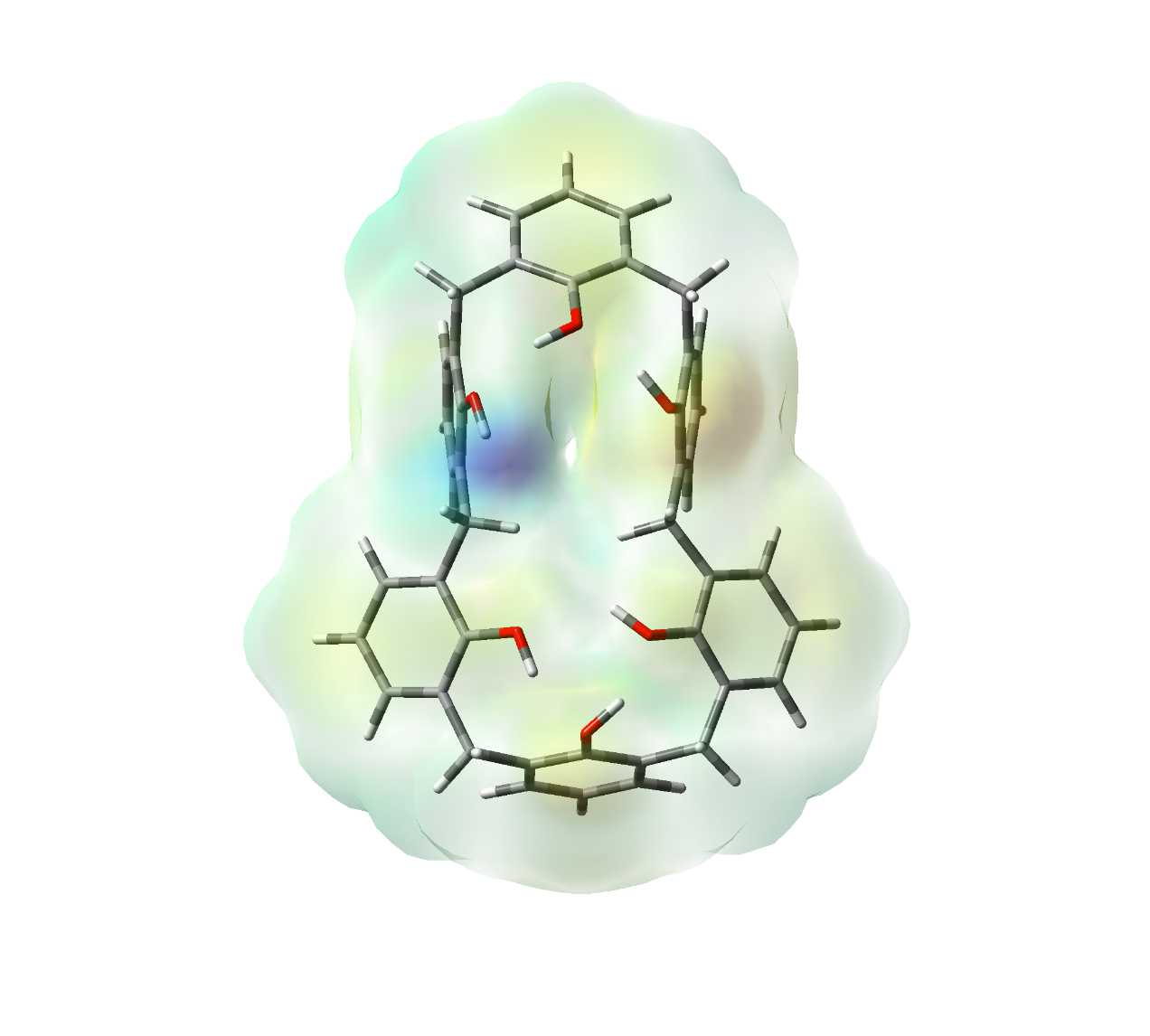


A

D


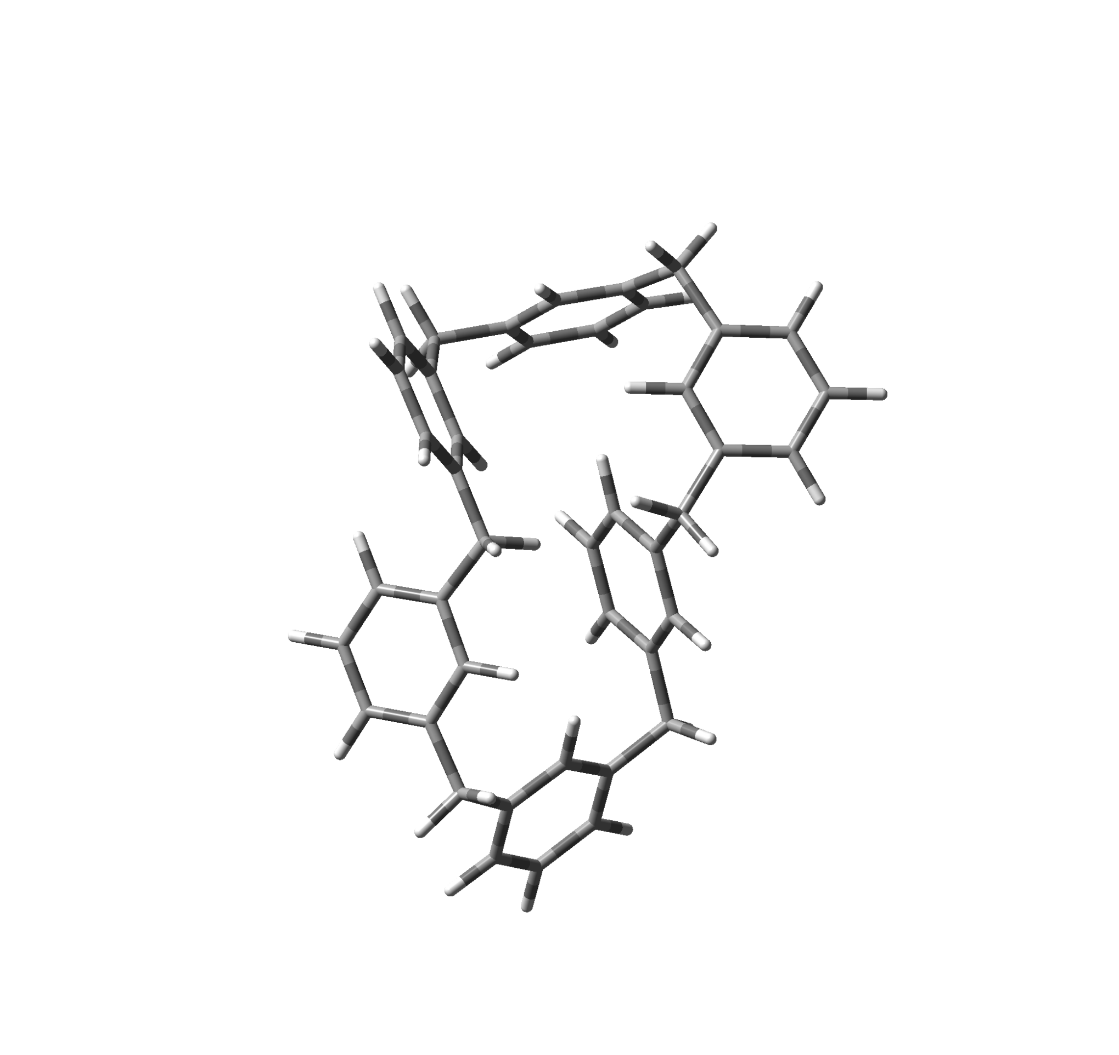

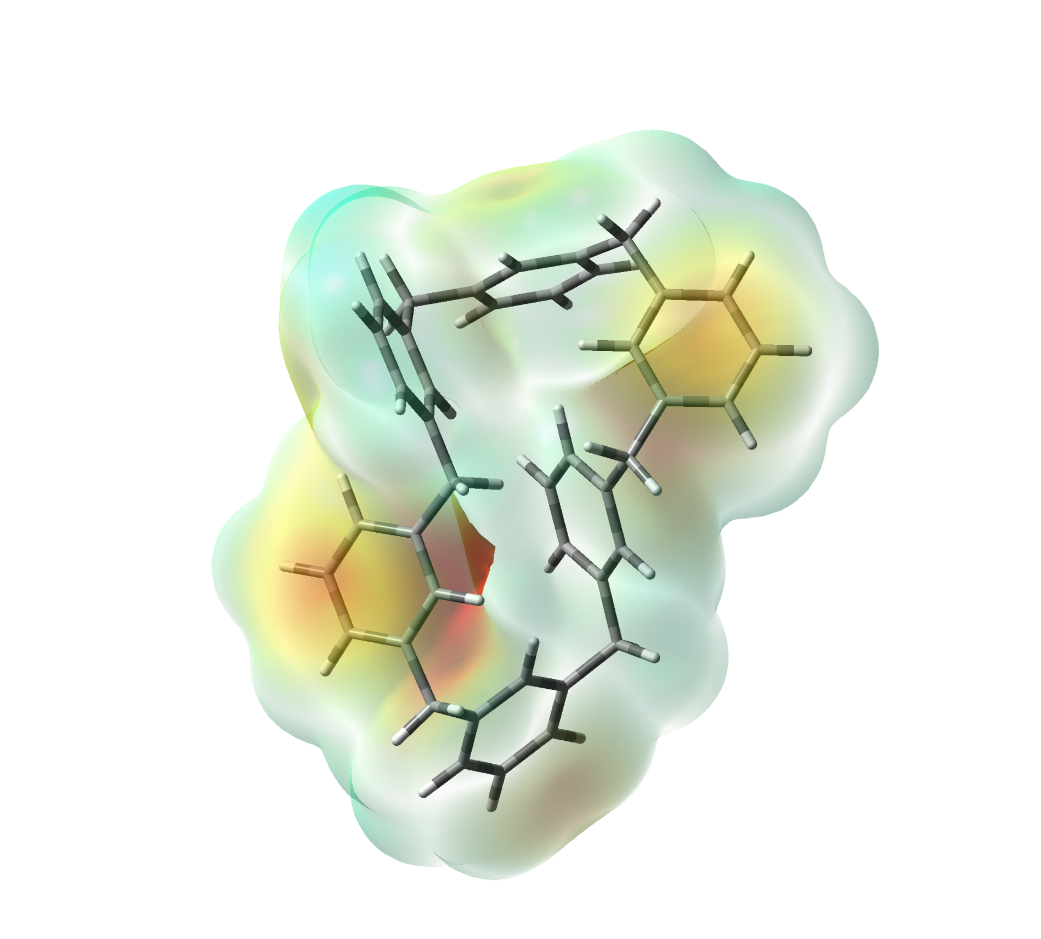

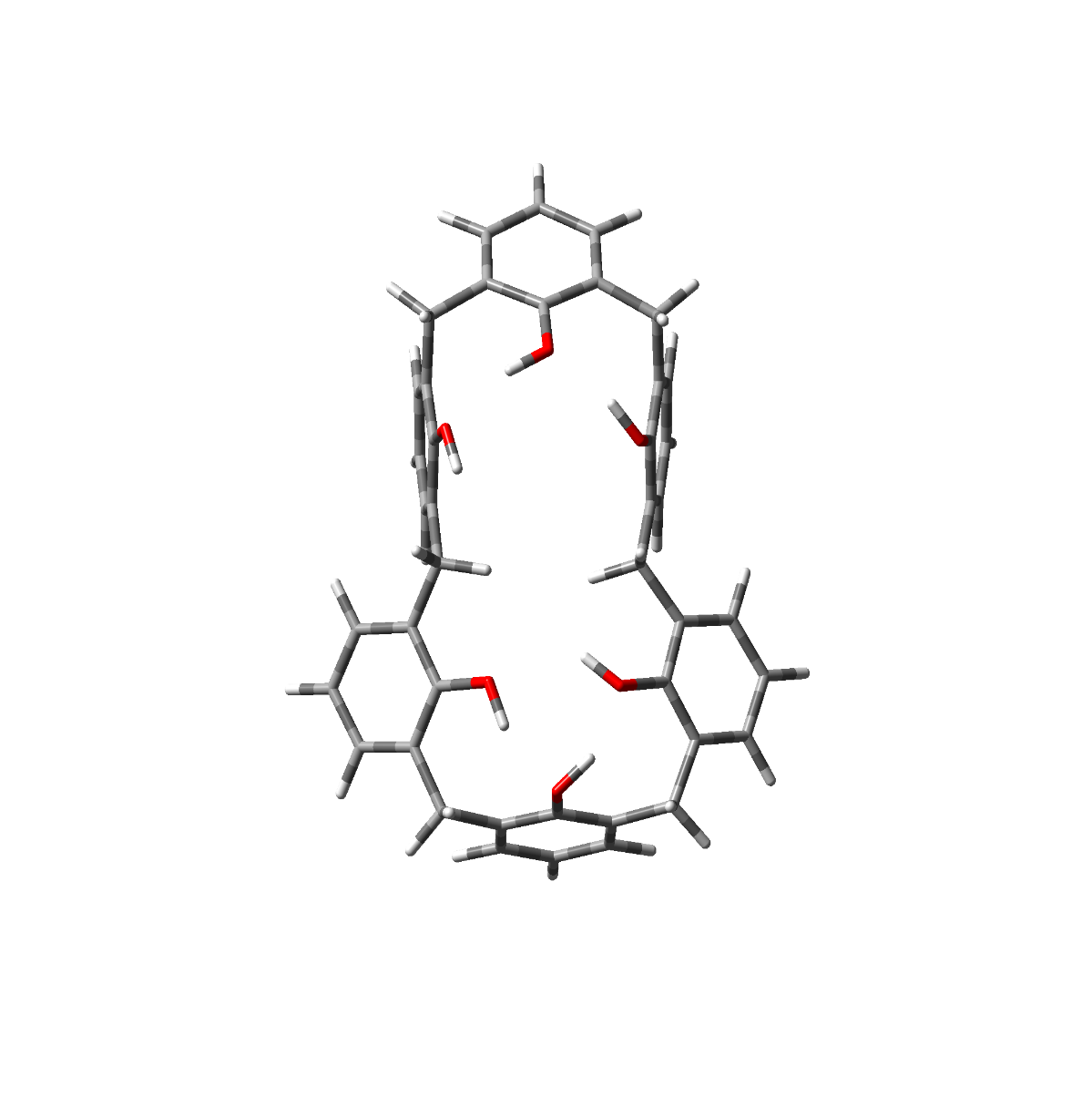


B

C

1.894

1.838

2.260

1.789

1.807

Figure S1: Panels A and B show the most energetically stable optimized geometrical structures for NCX6 and CX6 respectively as obtained in this work. Key structural parameters are reported in Å. Panels C and D show molecular electrostatic potential surfaces for NCX6 and CX6 respectively as obtained from the gas phase optimized structures using cubegen utility in the Gaussian 16 package. The color code indicates electron rich (red) and electron deficient sites (blue).


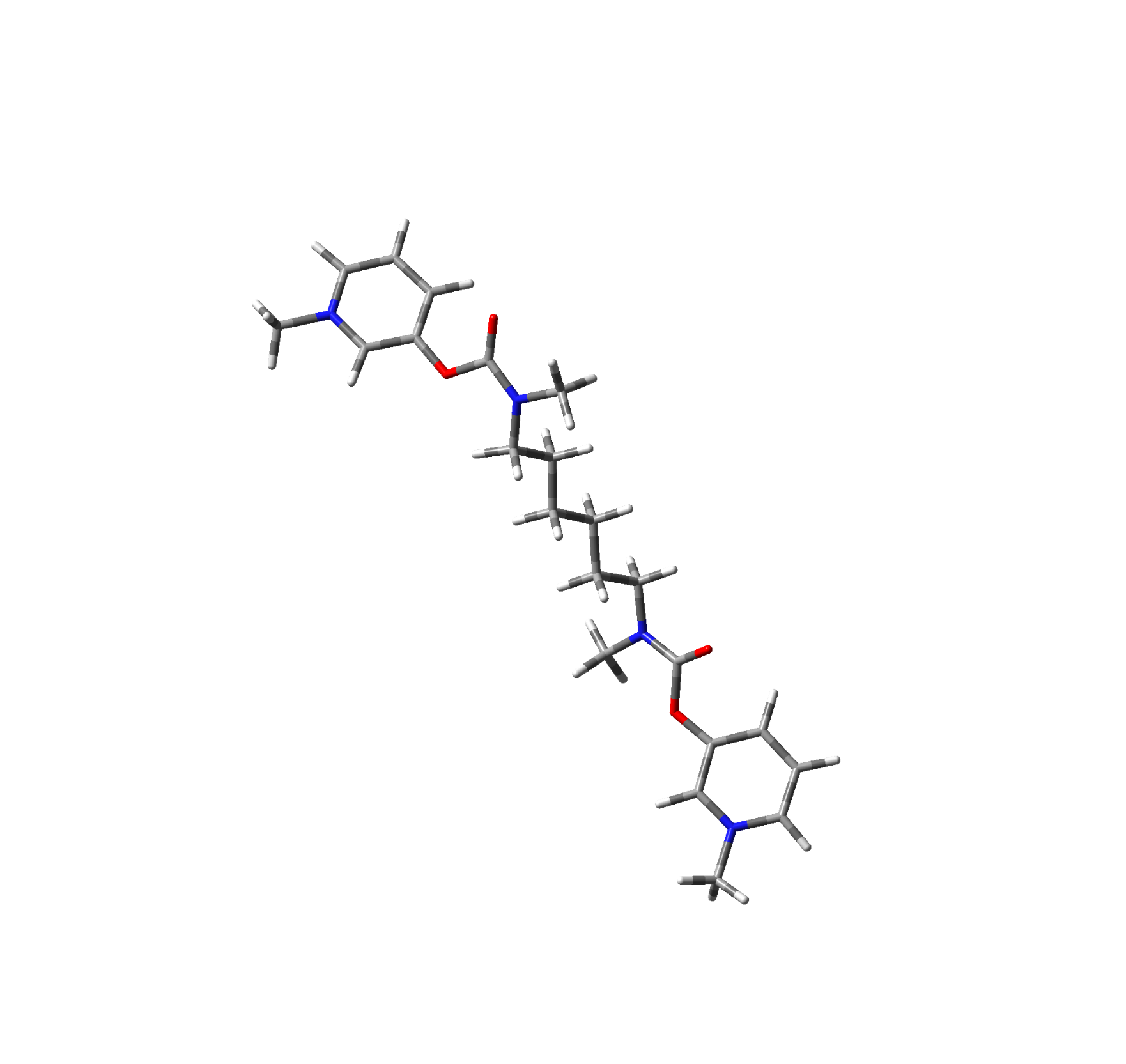

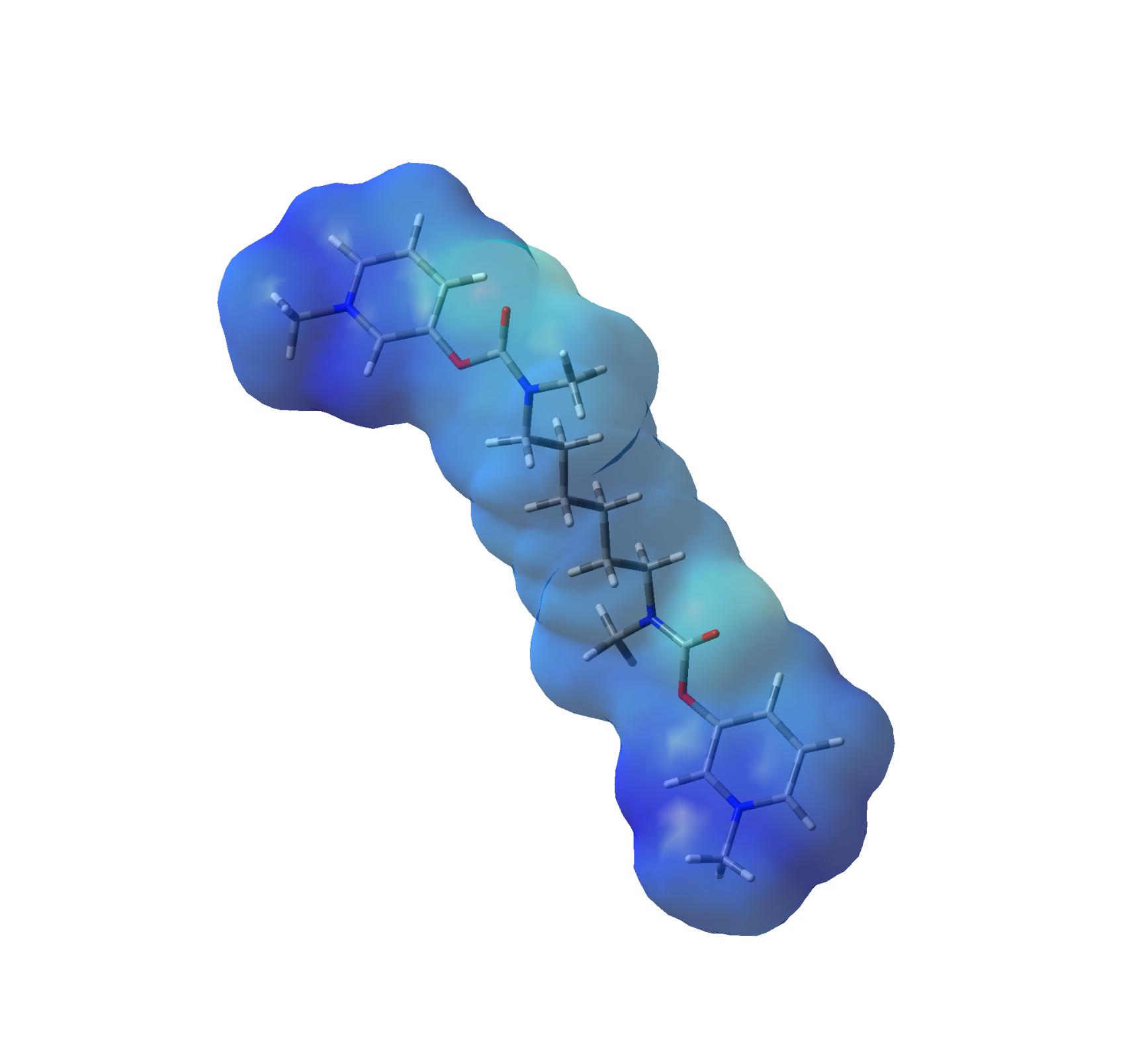

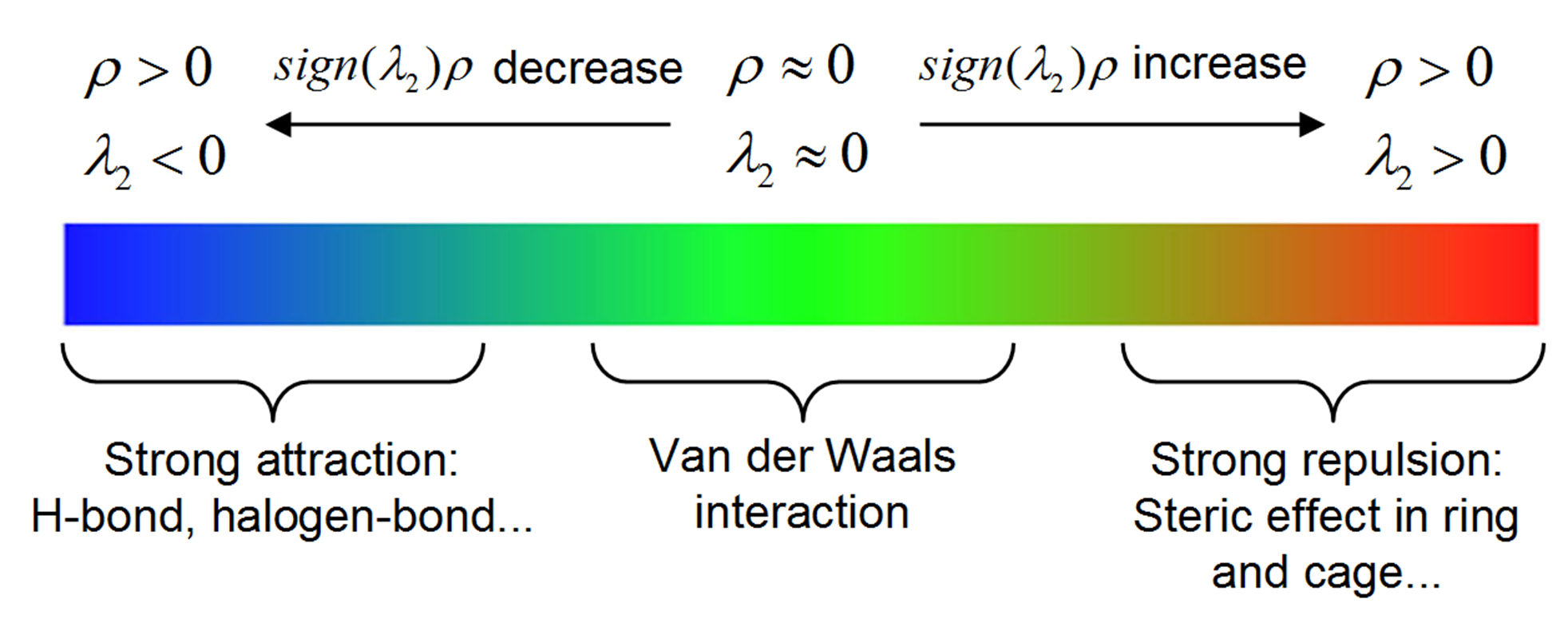


A

B

Figure S2: Panel A shows the most energetically stable optimized geometrical structure of DG as obtained in this work. Panel B shows molecular electrostatic potential surface for DG as obtained from the gas phase optimized structures using cubegen utility in the Gaussian 16 package. The color code indicates electron rich (red) and electron deficient sites (blue).


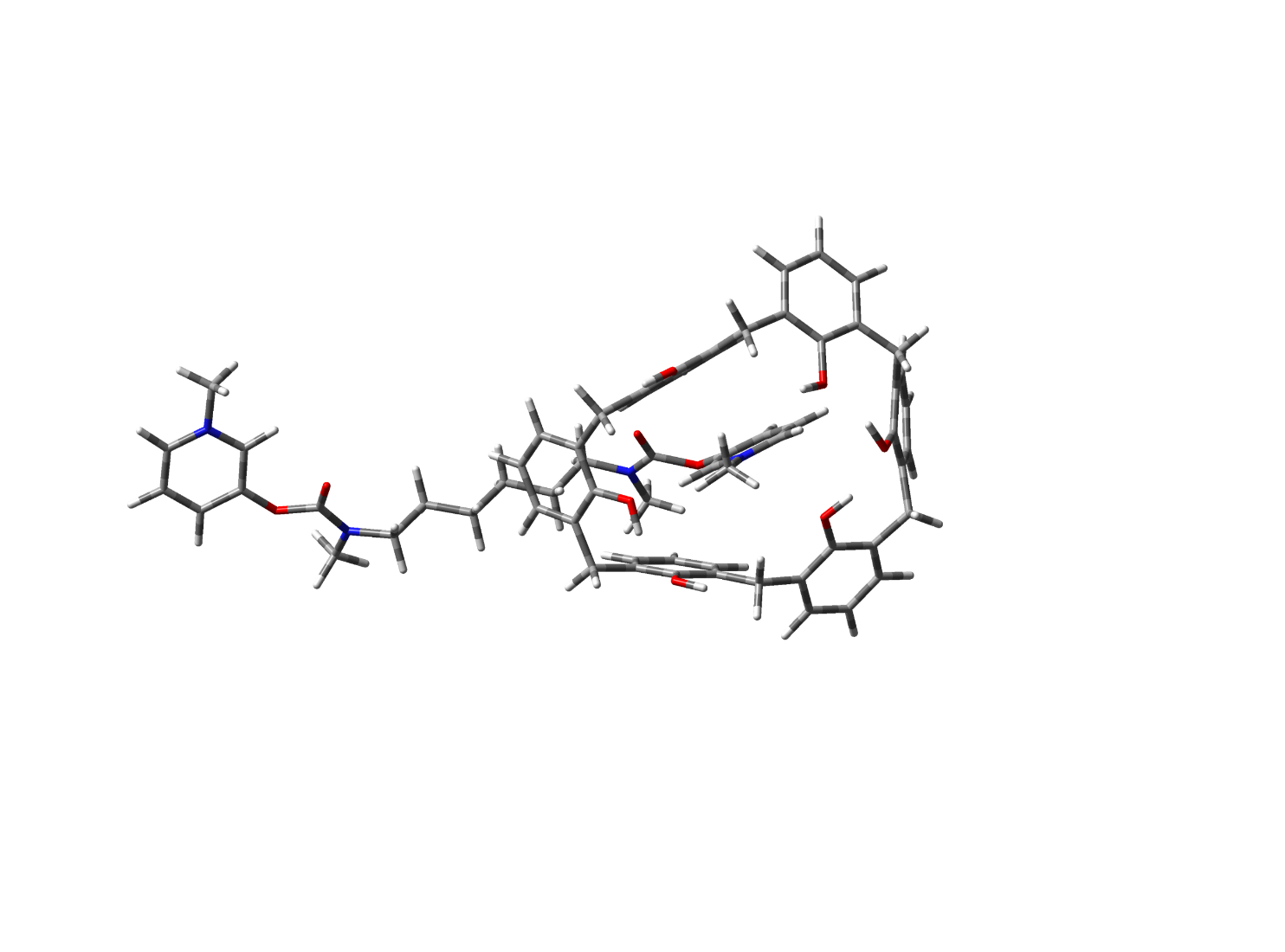


A

C

B

D


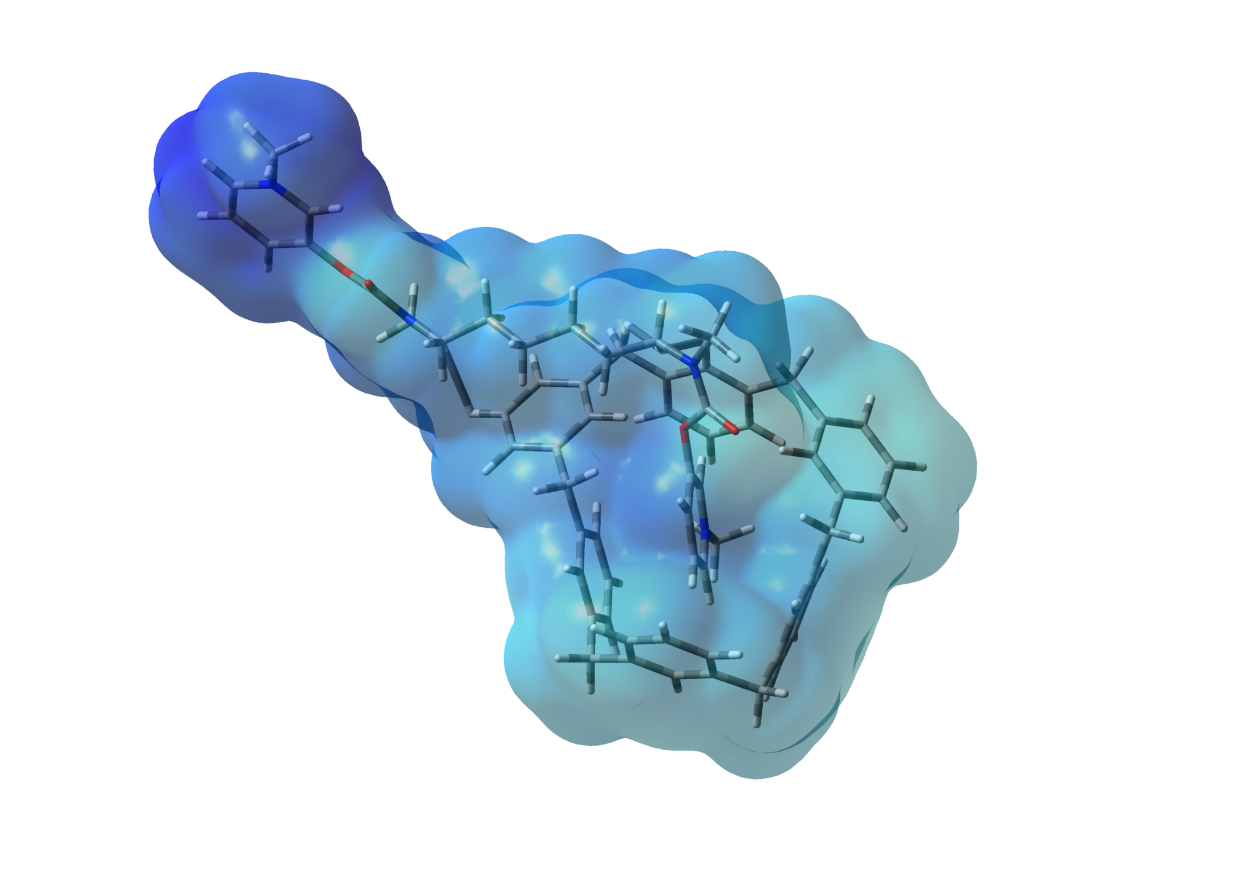

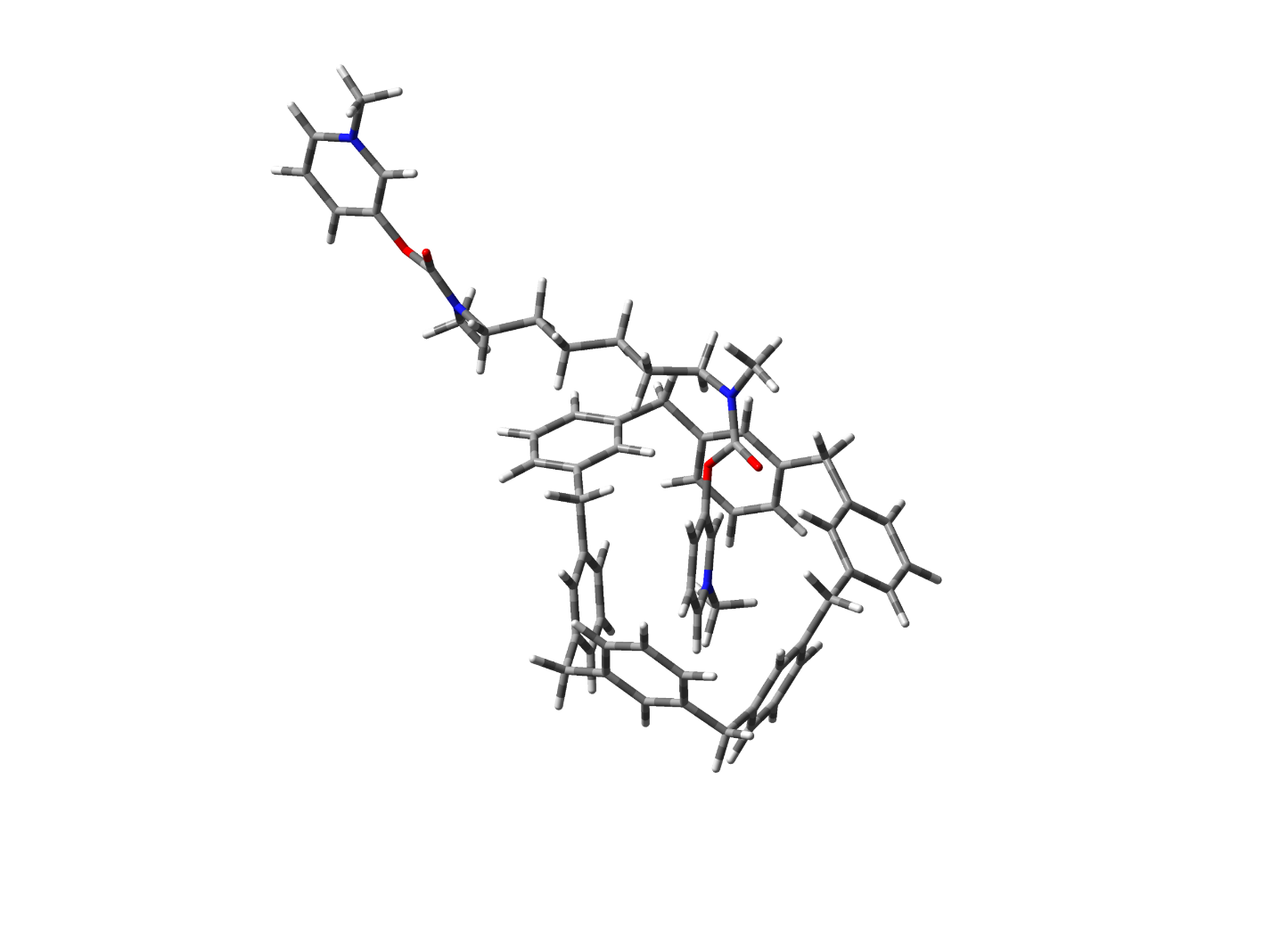

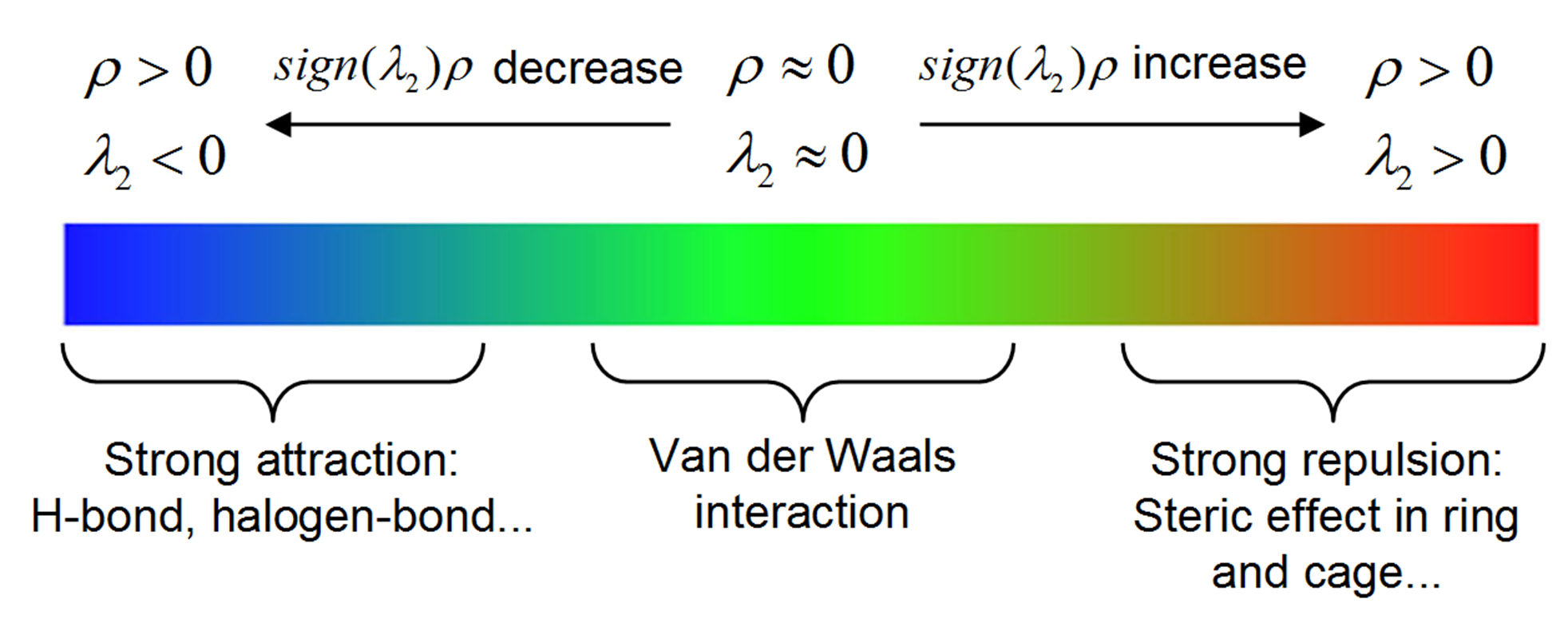

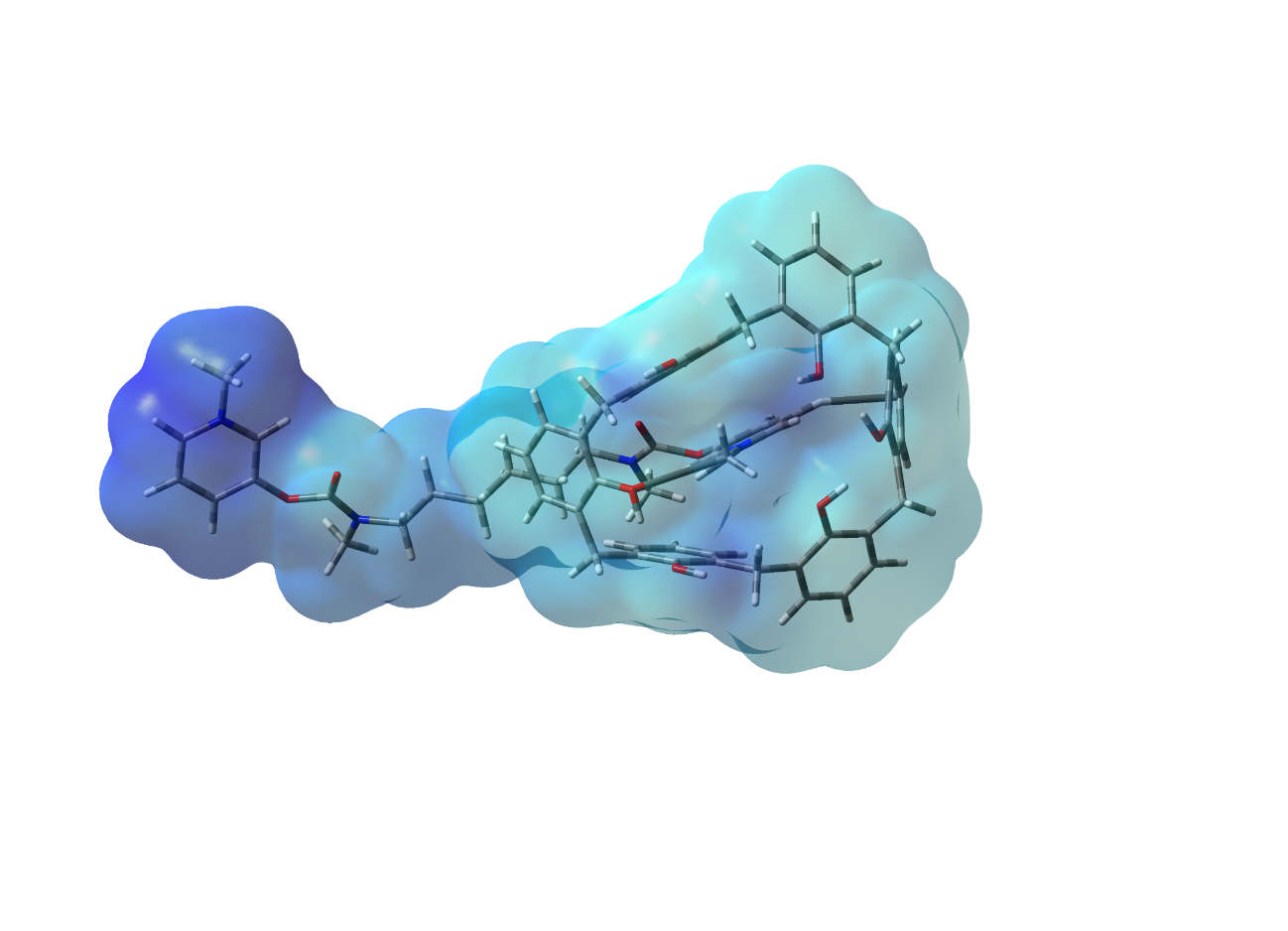


1.924

1.876

1.904

Figure S3: Panels A and B show optimized geometrical structures for 1:1 DG complexes with NCX6 and CX6 respectively as obtained using the B97D/6-31G(d,p) level of theory (6-31+G(d,p) was used for oxygen atoms). Key structural parameters are reported in Å. Panels C and D show molecular electrostatic potential surfaces as obtained from the gas phase optimized structures using cubegen utility in the Gaussian 16 package. The color code indicates electron rich (red) and electron deficient sites (blue).


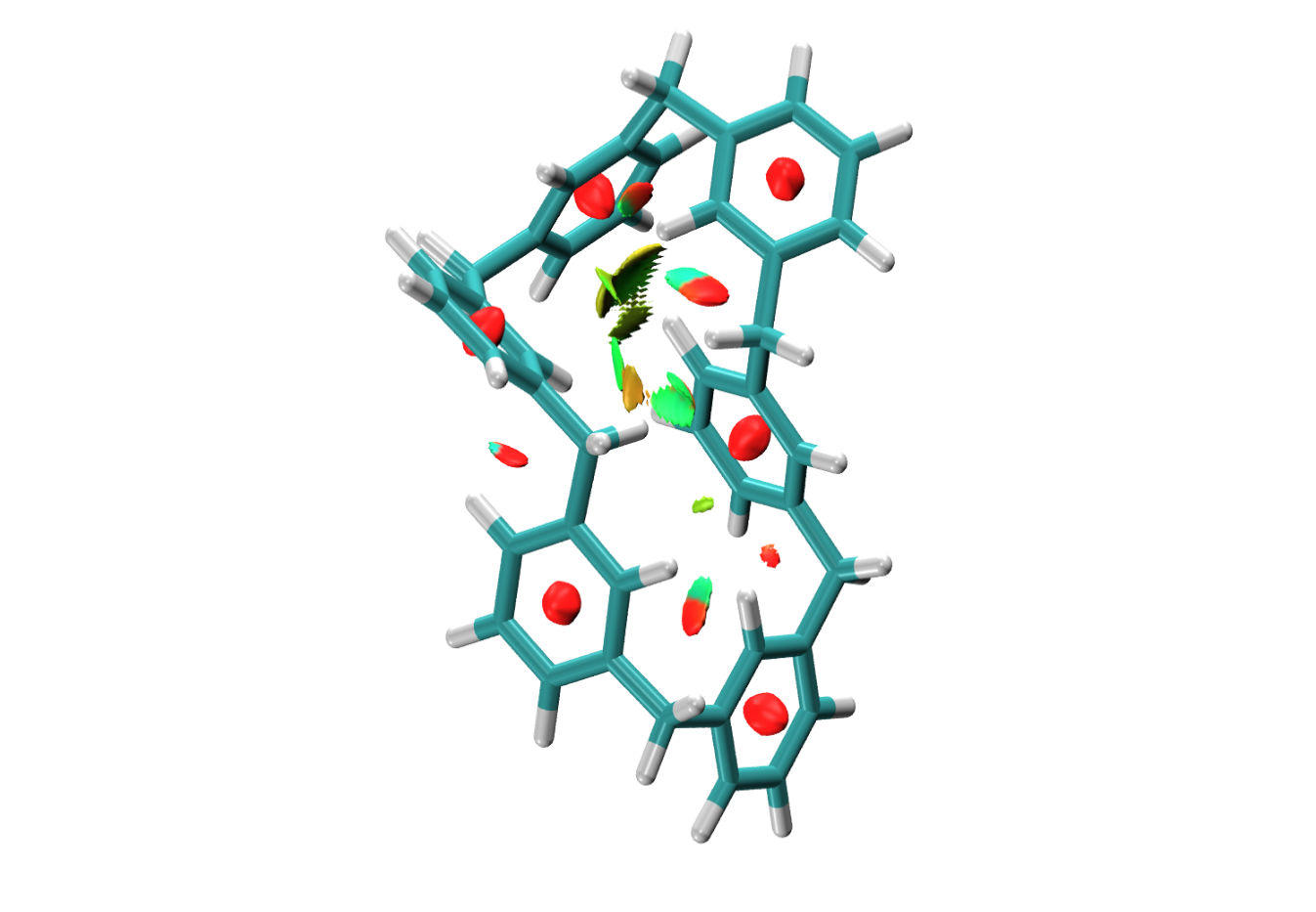

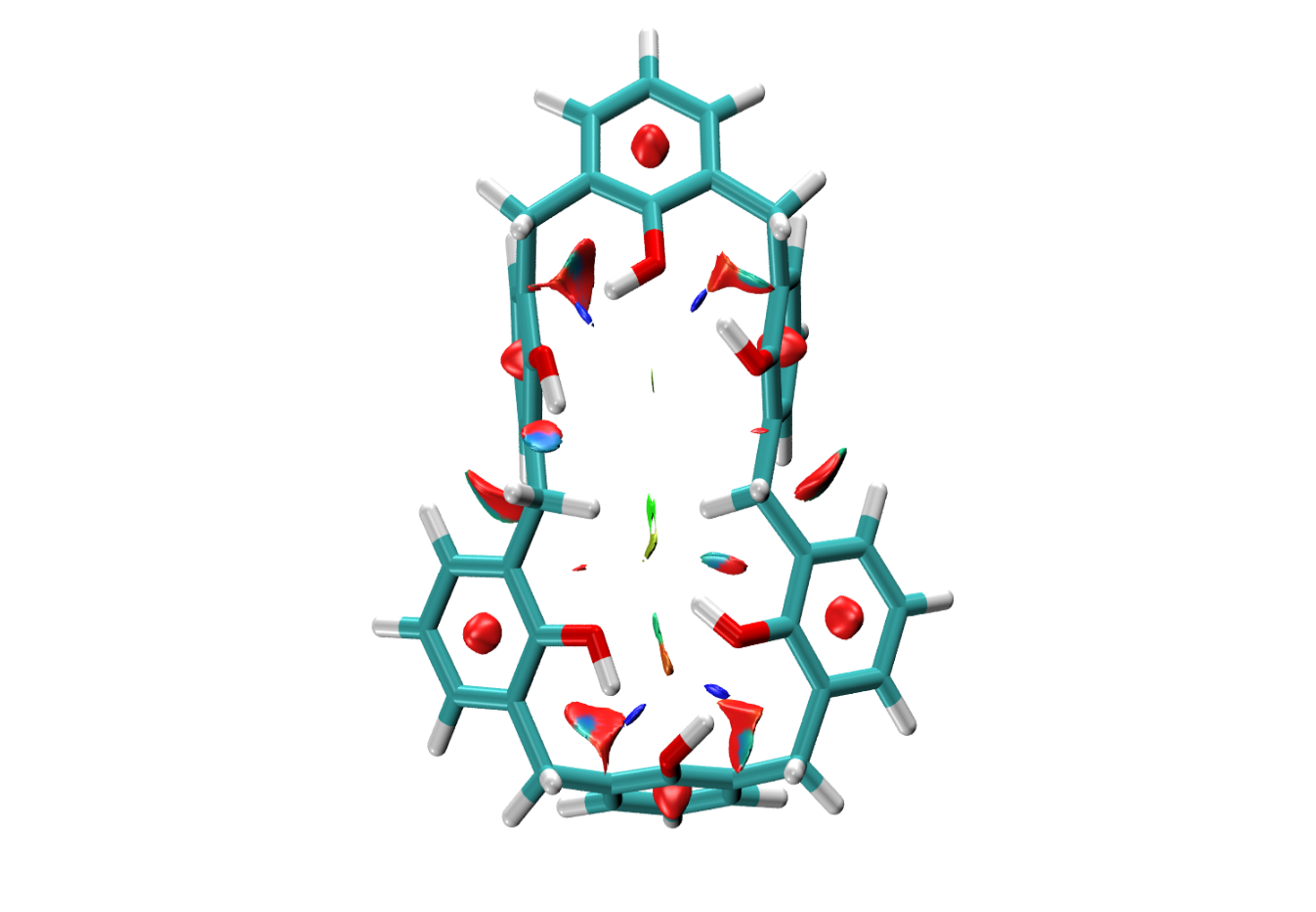


D

E


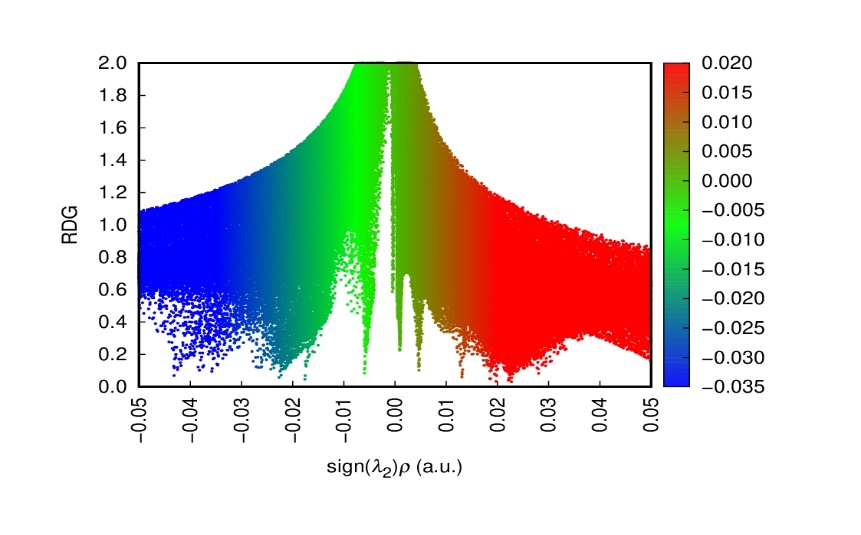

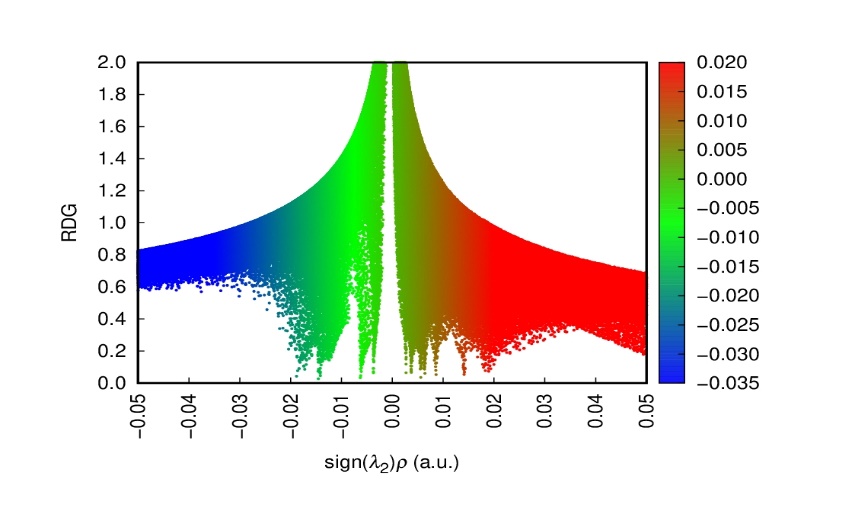

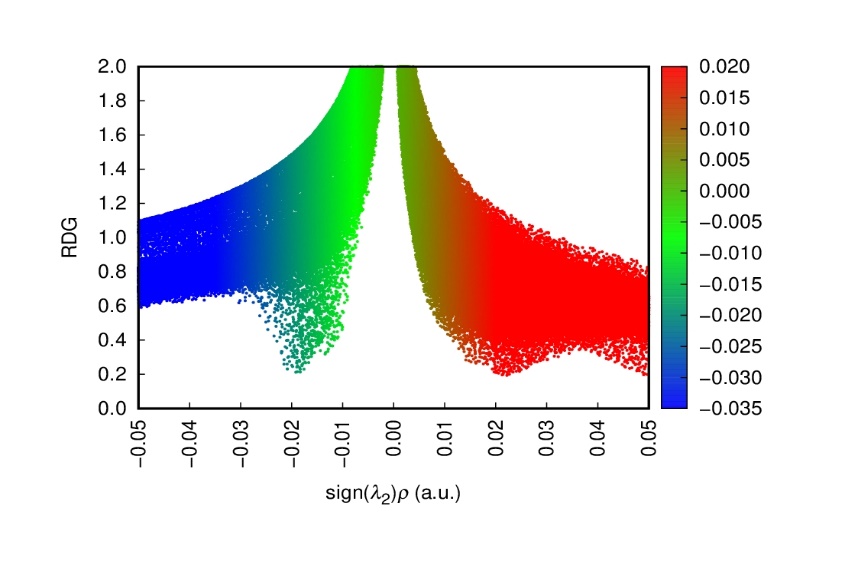


F

C

B

A


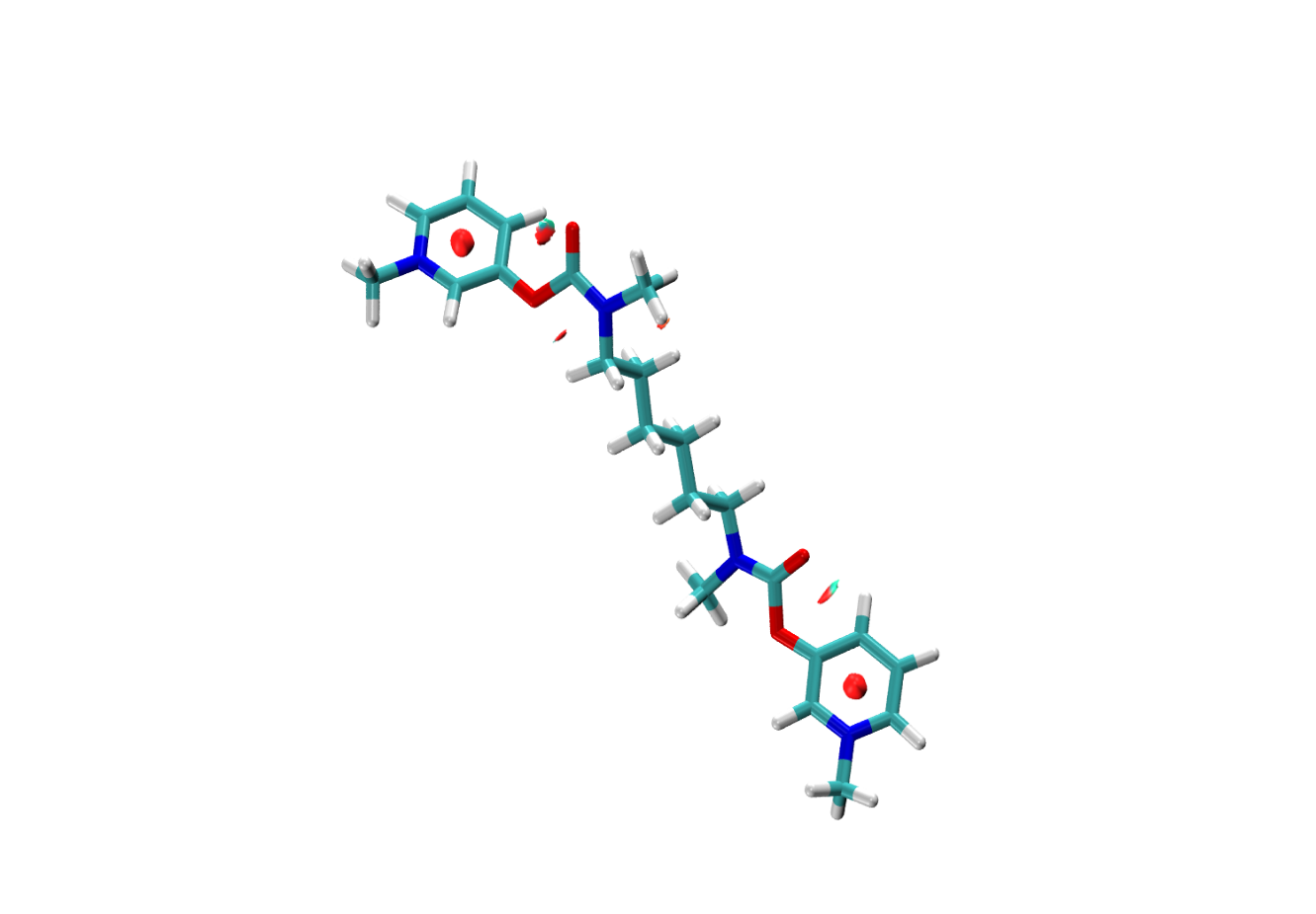


Figure S4: Panels A, B and C show NCI rendering for NCX6, CX6 and DG. Panels D, E and F show their respective reduced density gradient plots


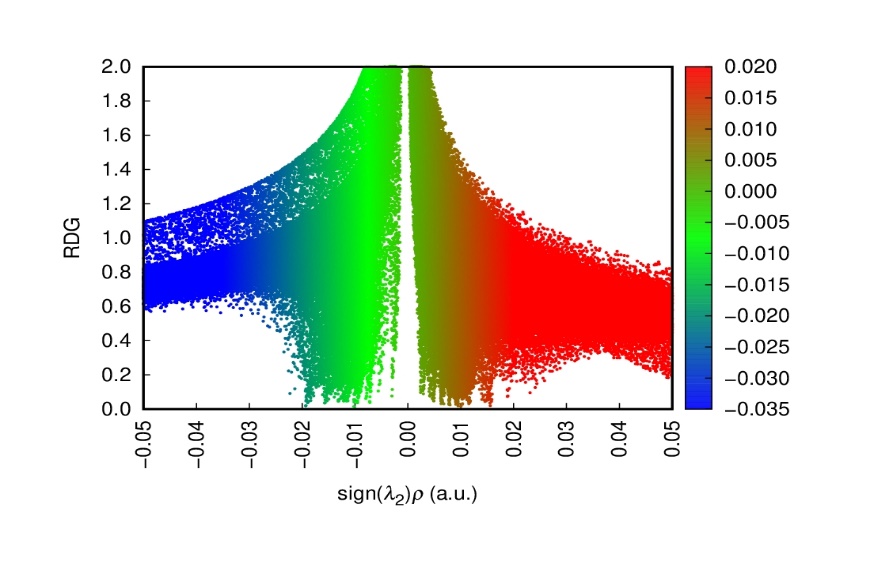

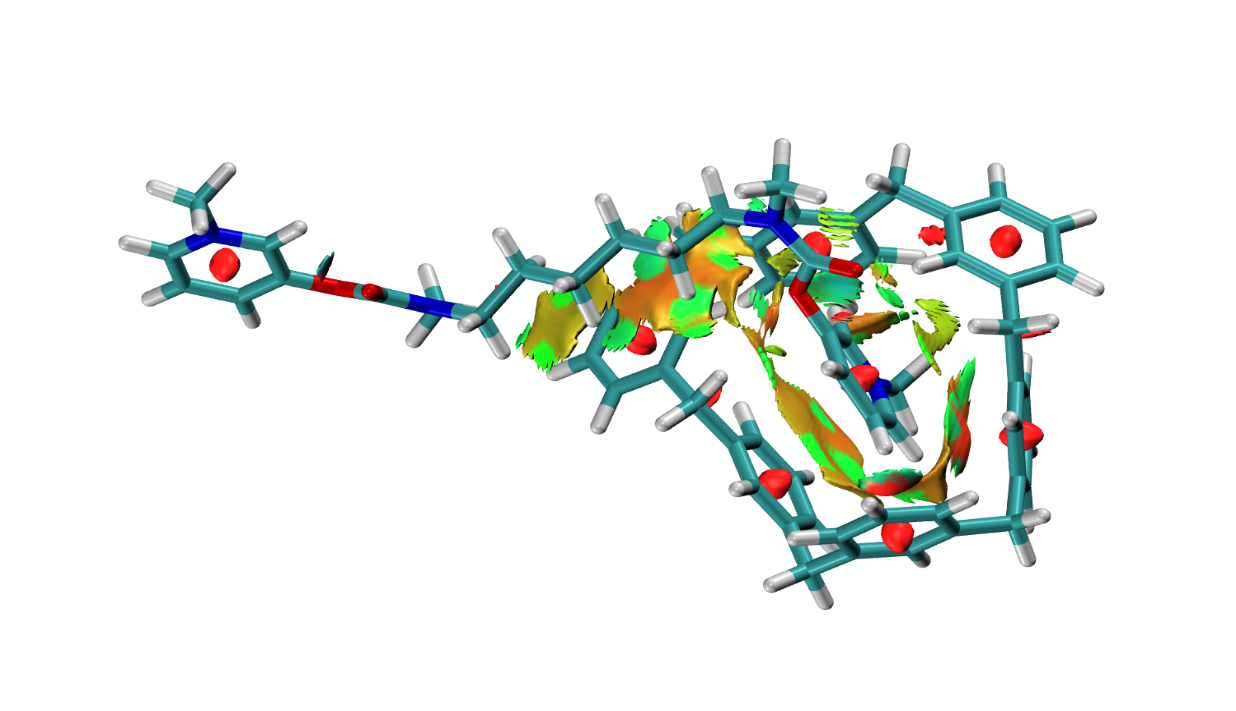


A

C


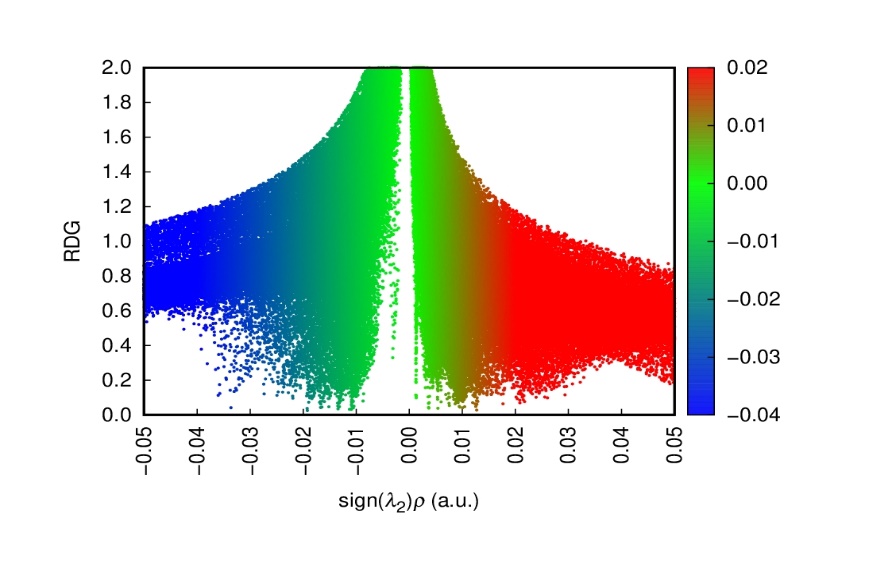

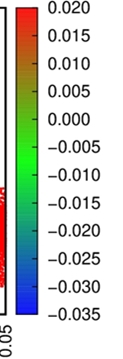


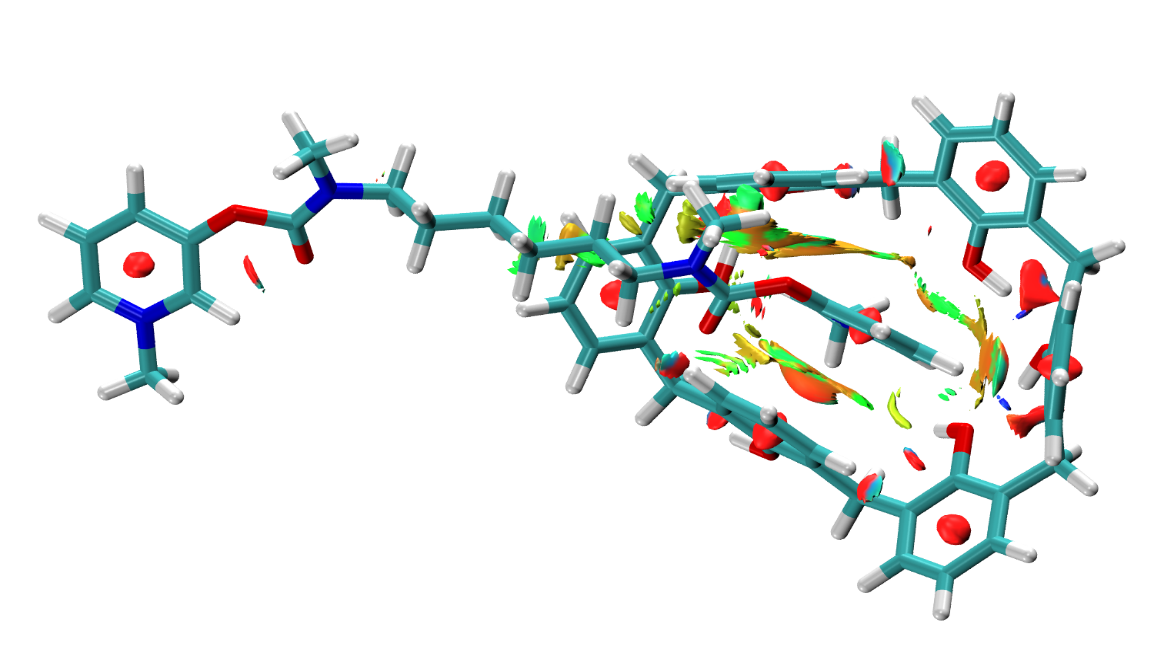


B

D

Figure S5: Panels A and B show NCI rendering for 1:1 complexes of DG with NCX6 and CX6 respectively. Panels C and D show their respective reduced density gradient plots


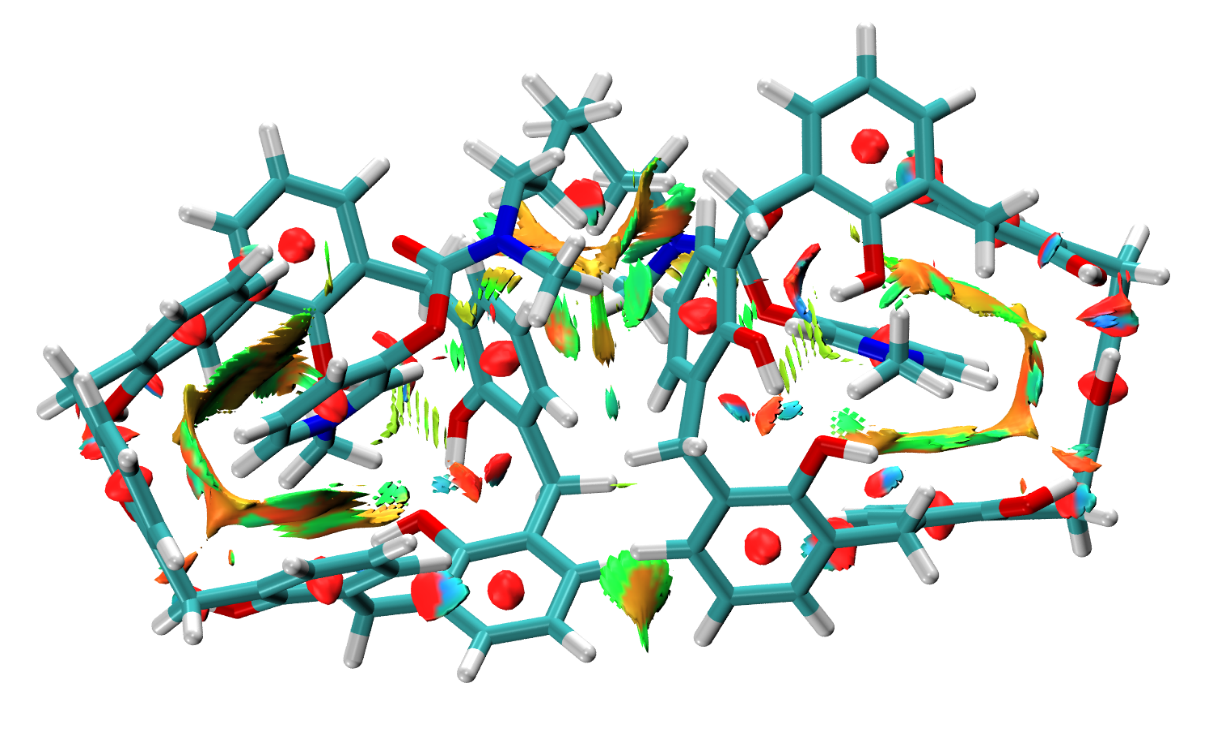

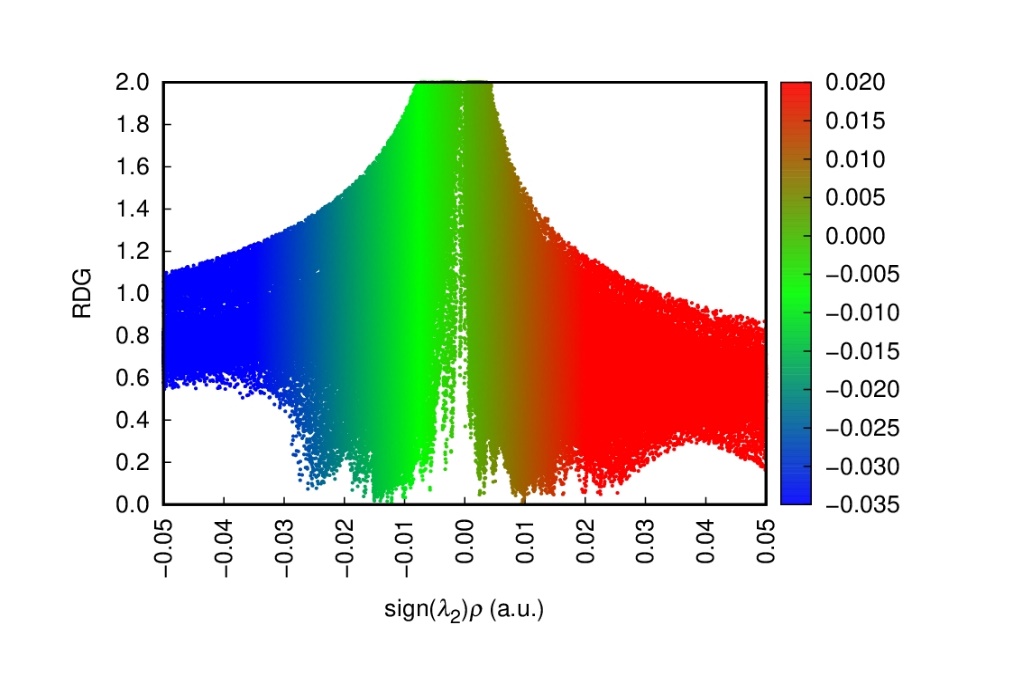

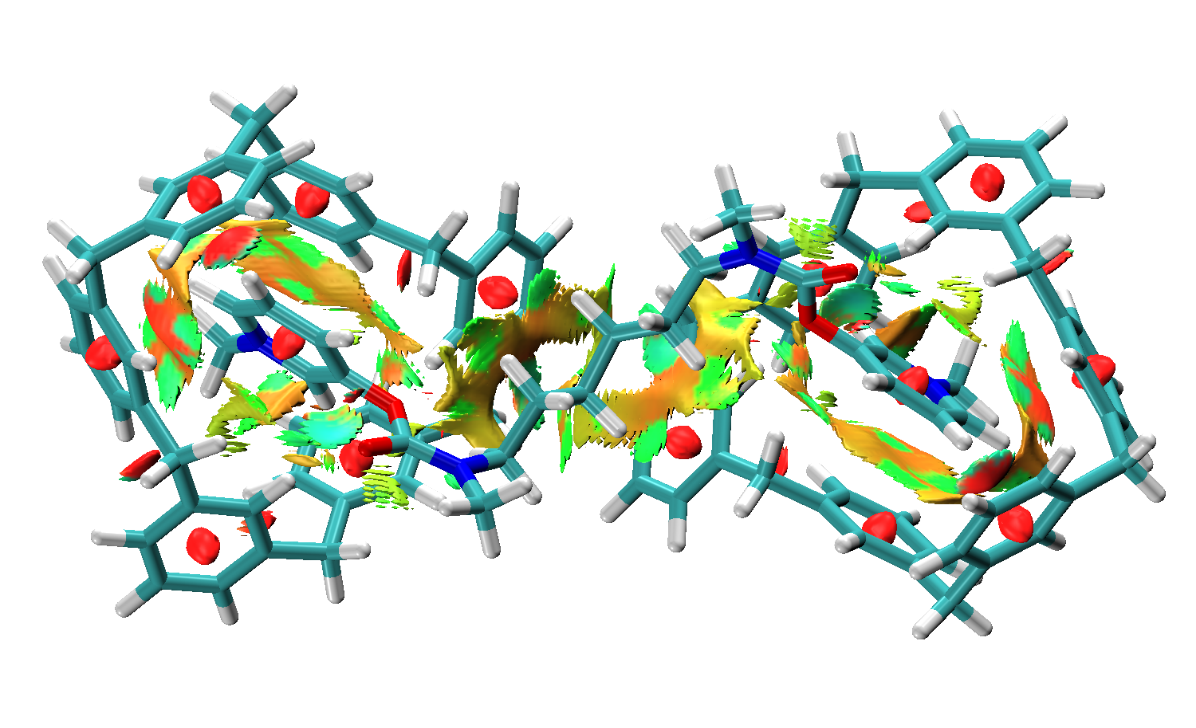

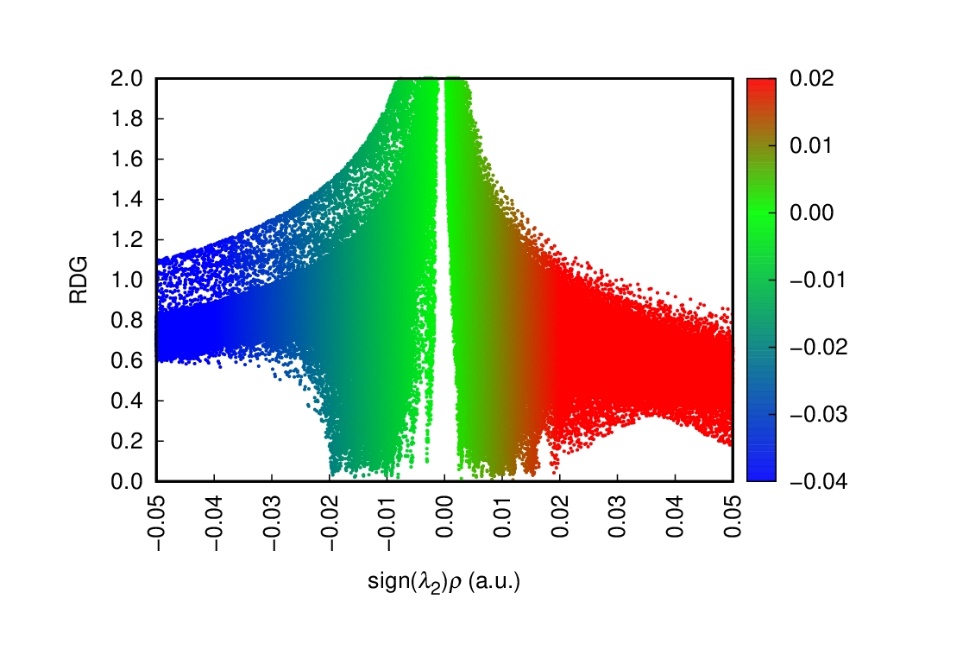

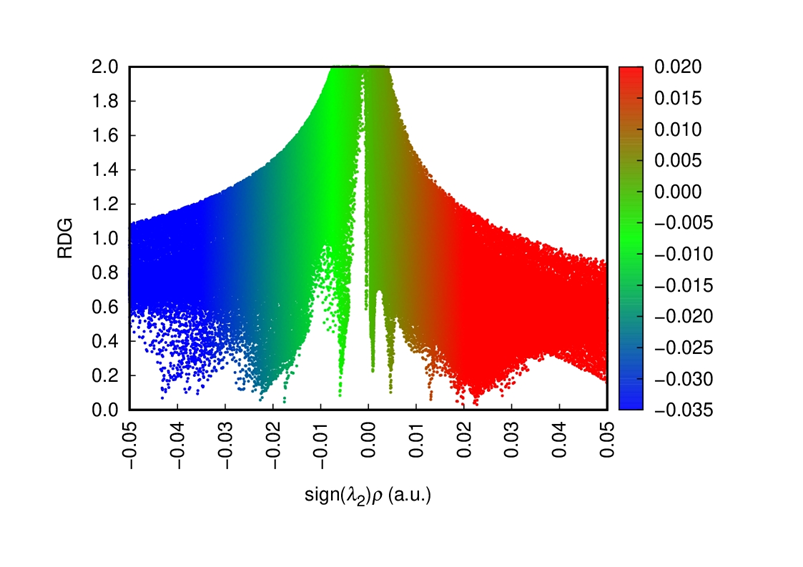


Figure S6: Panels A and B show NCI rendering for complexes of 1:2 DG with NCX6 and CX6 respectively. Panels C and D show their respective reduced density gradient plots

A

B

C

D


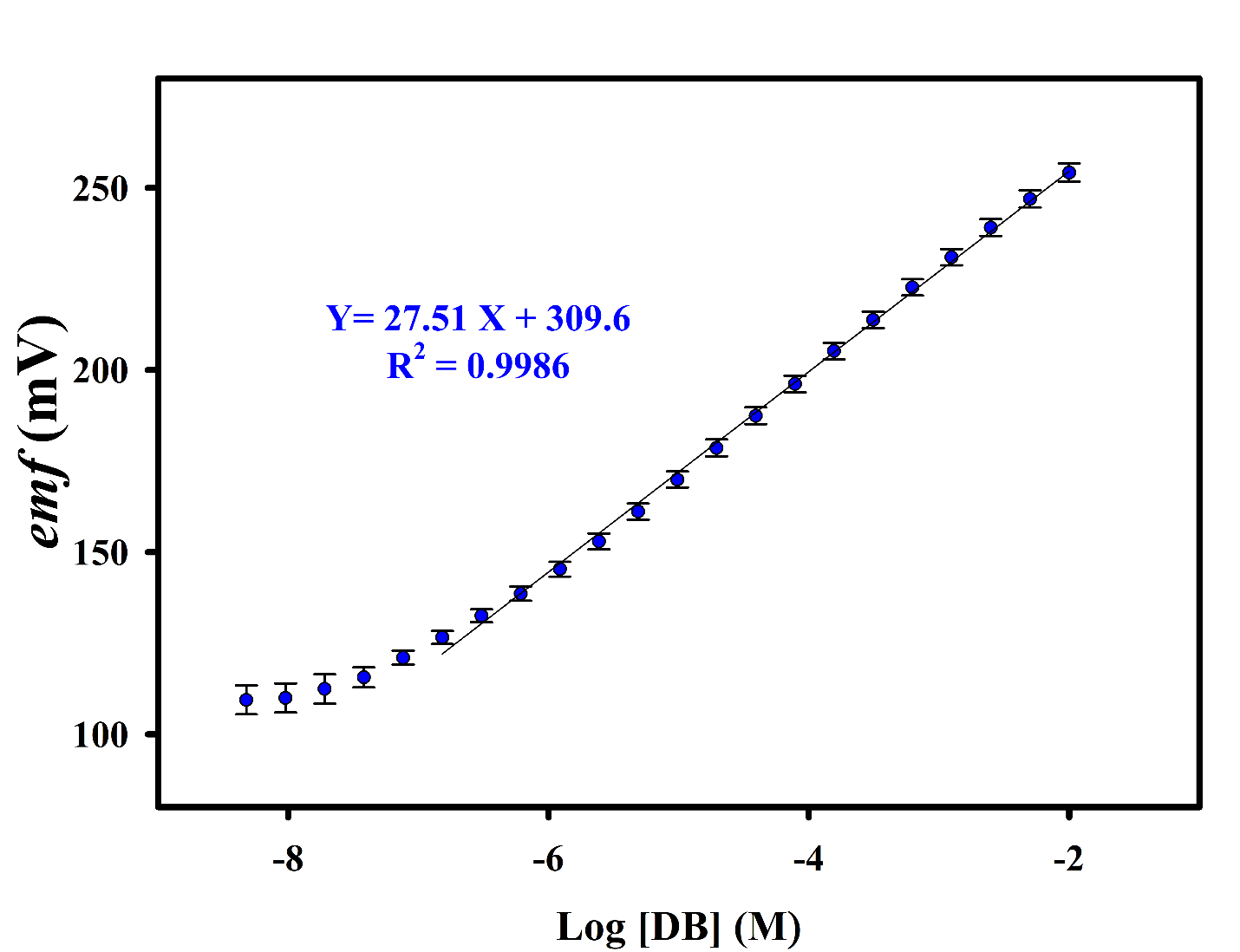


Figure S7: Calibration plot of DB solution (10 mM initial concentration) in phosphate buffer solution (pH = 7.0) at room temperature as a function of DB concentrations obtained via the successive dilution method by measuring the emf with repeated removal of an aliquot of the sample and addition of the corresponding phosphate buffer solution. Each point is the average obtained from six different screen-printed sensors. The error bars shown represent the RSD values of the six measurements.


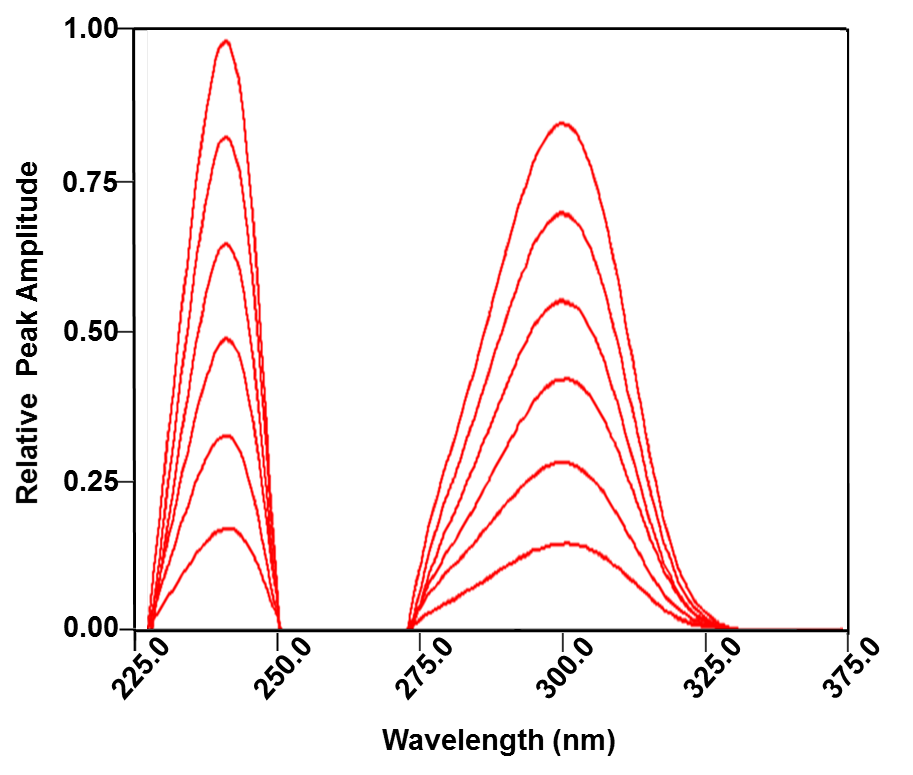


Figure S8: Difference absorption spectra of 10-60 μg/ml DB degradation product, TMPH, against the same concentration of intact DB as blank in phosphate buffer (pH 7).


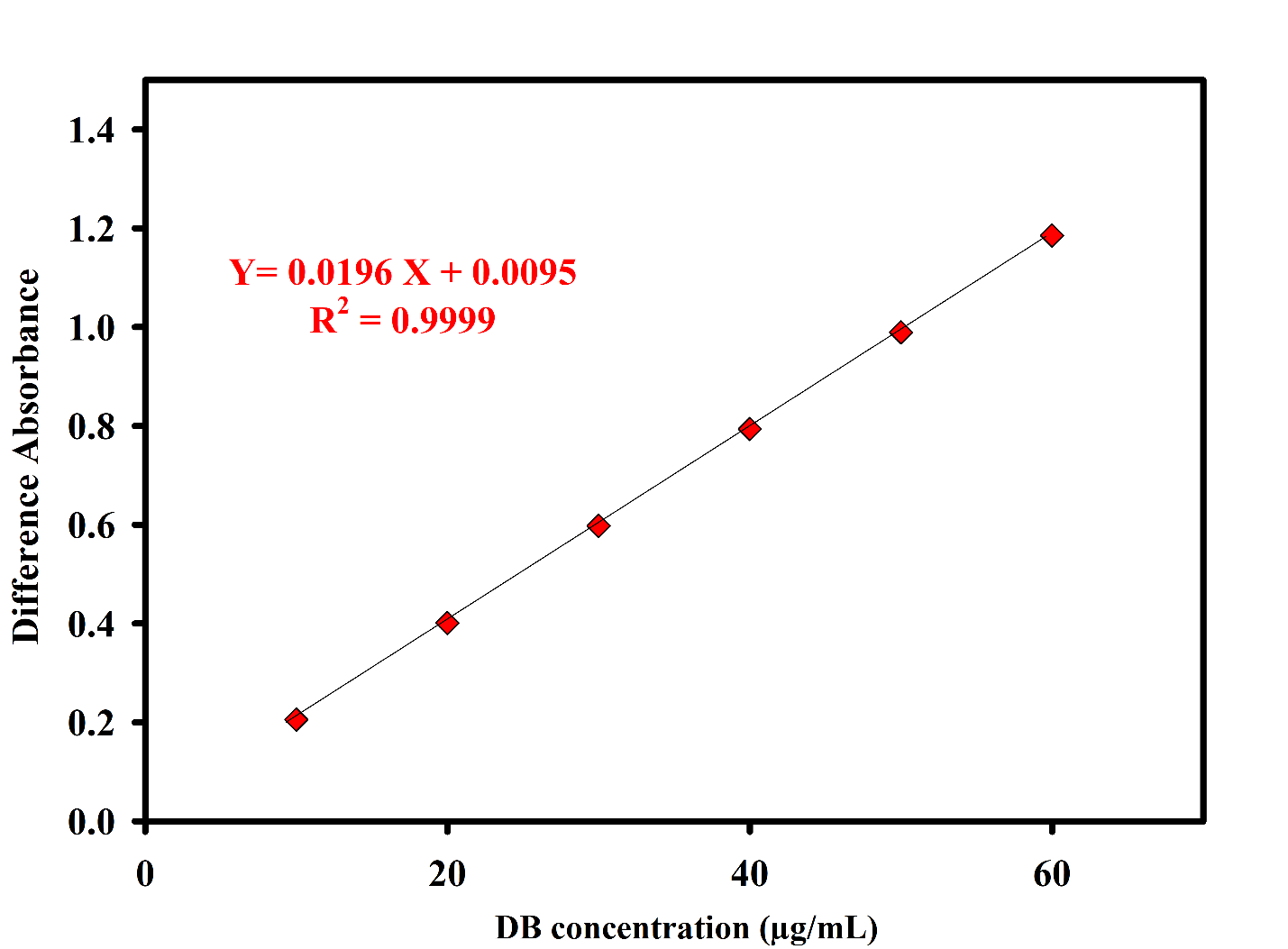


Figure S9: Linearity of difference absorbance of the alkaline degradate of distigmine bromide to the corresponding concentration of distigmine bromide, at 320 nm.


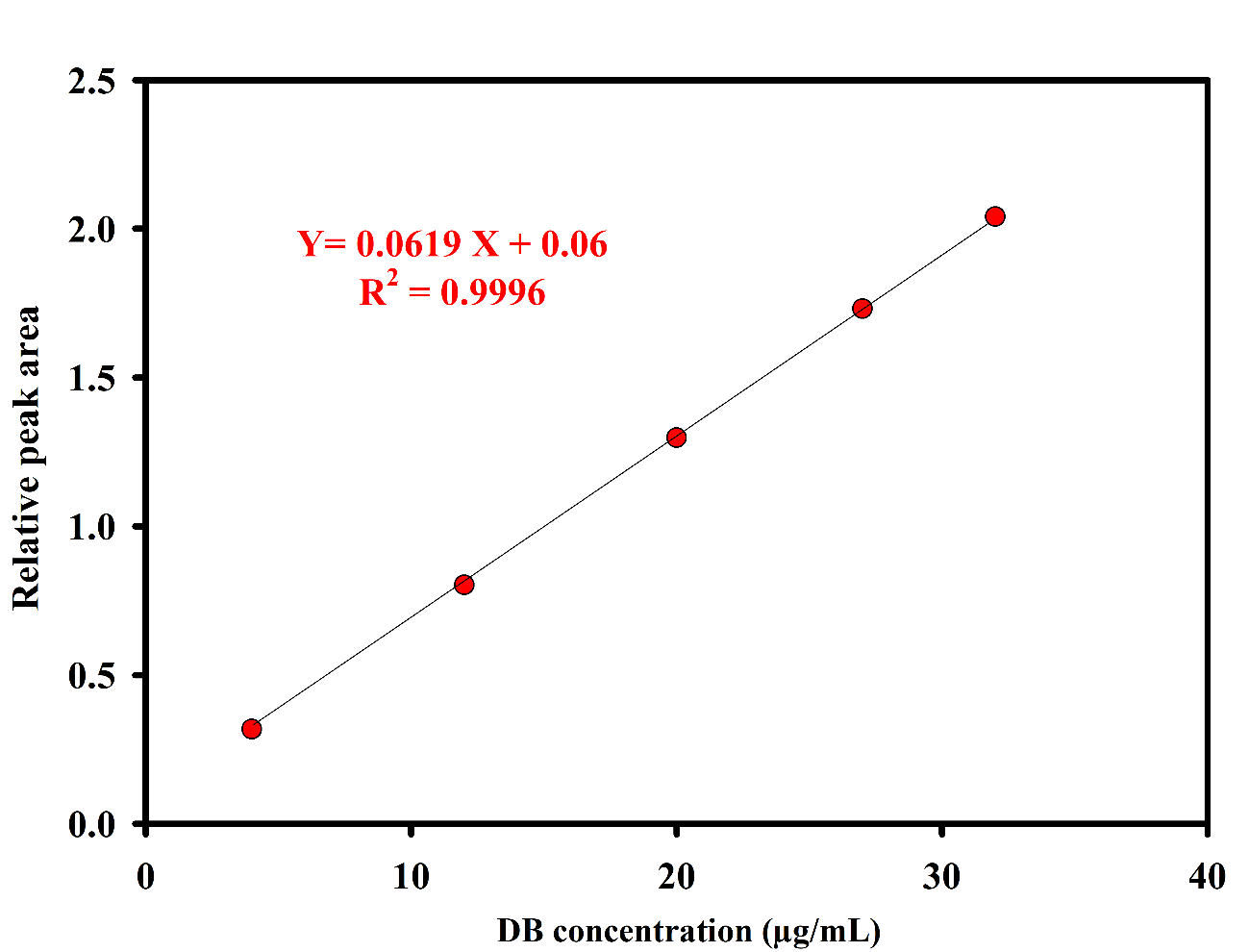


Figure S10: HPLC calibration plot for DB shows a linear relationship, R^2^ = 0.9996, between relative areas under DB peaks observed at retention time (T_R_) of 4.45 ± 0.02 min at 220 nm as shown in Figure 7 and their corresponding concentrations of DB (4 – 32 µg/mL).”


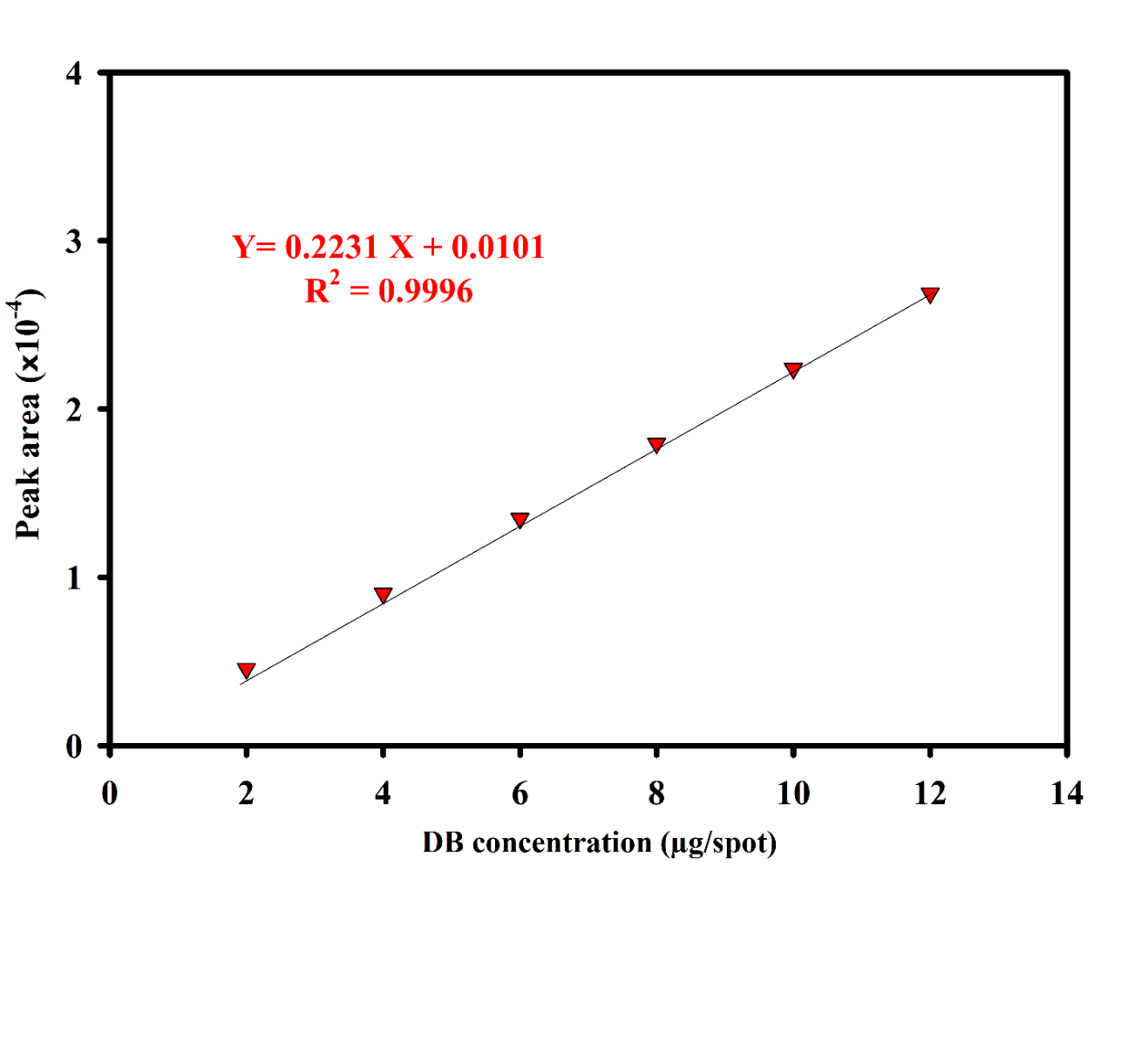


Figure S11: TLC densitometric calibration curve for DB shows a linear relationship, R^2^ = 0.9996, between integrated peak area at 230 nm and the corresponding concentrations of DB (2.– 12 µg/spot).

Movie S12: Two dimensional representation of UV absorption spectra of distigmine in alkaline buffer (BRB of pH 10) taken over 3 hours of hydrolysis at temp 25 ºC, during which the signal due the distigmine observed at 270 nm was gradually reduced and the signals due to the hydrolysis product, THMP, at 252 and 320 nm were gradually increased.
